# An empirical evaluation of two-stage species tree inference strategies using a multilocus dataset from North American pines

**DOI:** 10.1186/1471-2148-14-67

**Published:** 2014-03-29

**Authors:** Michael DeGiorgio, John Syring, Andrew J Eckert, Aaron Liston, Richard Cronn, David B Neale, Noah A Rosenberg

**Affiliations:** 1 Department of Biology, Pennsylvania State University, University Park, PA 16802, USA; 2 Department of Biology, Linfield College, McMinnville, OR 97128, USA; 3 Section of Evolution and Ecology & Center for Population Biology, University of California, Davis, CA 95616, USA; 4 Department of Biology, Virginia Commonwealth University, Richmond, VA 23284, USA; 5 Department of Botany and Plant Pathology, Oregon State University, Corvallis, OR 97331, USA; 6 Pacific Northwest Research Station, USDA Forest Service, Corvallis, OR 97331, USA; 7 Department of Plant Sciences, University of California, Davis, CA 95616, USA; 8 Department of Biology, Stanford University, Stanford, CA 94305, USA

## Abstract

**Background:**

As it becomes increasingly possible to obtain DNA sequences of orthologous genes from diverse sets of taxa, species trees are frequently being inferred from multilocus data. However, the behavior of many methods for performing this inference has remained largely unexplored. Some methods have been proven to be consistent given certain evolutionary models, whereas others rely on criteria that, although appropriate for many parameter values, have peculiar zones of the parameter space in which they fail to converge on the correct estimate as data sets increase in size.

**Results:**

Here, using North American pines, we empirically evaluate the behavior of 24 strategies for species tree inference using three alternative outgroups (72 strategies total). The data consist of 120 individuals sampled in eight ingroup species from subsection *Strobus* and three outgroup species from subsection *Gerardianae*, spanning ∼47 kilobases of sequence at 121 loci. Each “strategy” for inferring species trees consists of three features: a species tree construction method, a gene tree inference method, and a choice of outgroup. We use multivariate analysis techniques such as principal components analysis and hierarchical clustering to identify tree characteristics that are robustly observed across strategies, as well as to identify groups of strategies that produce trees with similar features. We find that strategies that construct species trees using only topological information cluster together and that strategies that use additional non-topological information (e.g., branch lengths) also cluster together. Strategies that utilize more than one individual within a species to infer gene trees tend to produce estimates of species trees that contain clades present in trees estimated by other strategies. Strategies that use the minimize-deep-coalescences criterion to construct species trees tend to produce species tree estimates that contain clades that are not present in trees estimated by the Concatenation, RTC, SMRT, STAR, and STEAC methods, and that in general are more balanced than those inferred by these other strategies.

**Conclusions:**

When constructing a species tree from a multilocus set of sequences, our observations provide a basis for interpreting differences in species tree estimates obtained via different approaches that have a two-stage structure in common, one step for gene tree estimation and a second step for species tree estimation. The methods explored here employ a number of distinct features of the data, and our analysis suggests that recovery of the same results from multiple methods that tend to differ in their patterns of inference can be a valuable tool for obtaining reliable estimates.

## Background

In phylogenetic studies, it has become increasingly common to sequence large numbers of individuals at many loci (e.g., [1-4]). While these multilocus datasets provide the potential to improve the accuracy of phylogeny inferences over large sets of taxa, for a variety of reasons, topologies of trees inferred at different loci might not match [5]. One source of this gene tree discordance is incomplete lineage sorting, the phenomenon in which sets of sampled lineages fail to coalesce in the population in which they are first capable of coalescing [6]. With incomplete lineage sorting, several species tree inference methods—including Concatenation [7], Democratic Vote Consensus [8], Greedy Consensus [9], Majority-Rule Consensus [9], Matrix Representation with Parsimony [10], and Minimize Deep Coalescences [11]—can be misled by discordance of gene trees across loci.

Numerous approaches have been used for estimating species tree topologies from multilocus sequence data. Consensus methods construct species tree topologies from gene trees according to deterministic rules applied to the input set of trees [12,13]. These methods take as input a set of gene trees produced from individual loci, allowing for separate input evolutionary histories at each locus. Because genetic lineages in different species sometimes have relatively few sequence differences, however, the information in a locus can be insufficient to accurately infer gene trees, thereby allowing incorrect gene trees to adversely influence the constructed species tree (e.g., [14,15]). Concatenation methods concatenate a set of multiple alignments and construct a tree from the concatenated alignment, treating the estimated “super-gene” tree as a species tree estimate [6,13,16]. Because concatenation combines all loci to form a single locus, and because different loci can have different evolutionary histories that are disregarded in the analysis of the concatenated alignment, the analysis of loci in this way can lead to incorrect species tree inferences [7]. Consensus and concatenation have in common that they are “two-stage” methods, in which species trees are inferred in two steps—inference of gene trees and then species tree inference for consensus, and inference of the super-gene tree followed by a conceptually substantial though methodologically trivial pronouncement that this tree is the species tree estimate for concatenation [6,12,13]. A third class of approaches can be labeled “single-stage” methods, in which species trees are inferred by simultaneously modeling the evolution of sequences among all sampled loci to output a species tree estimate [17-20]. These single-stage model-based methods often have desirable statistical properties, but because they typically explore large spaces of possibilities rather than algorithmically constructing estimated trees, they can be computationally intensive and applicable only to smaller datasets.

Properties of species tree inference methods can be examined using a variety of frameworks, including theory, simulations, and empirical assessments. Theoretical investigations are often concerned with limiting properties as the number of loci approaches infinity [9-11,15,21-27]. In-depth explorations of inference methods often rely on simulation studies, which are commonly used to investigate the performance of species tree inference methods on simulated multilocus datasets [10,28-32]. These theoretical and simulation-based studies have the advantage of knowing the true species tree, but the disadvantage that the scenarios they examine lack the complexity of empirical data.

An alternative approach is to evaluate methods on an empirical dataset in which the space of parameter values is defined by the evolutionary history of a group of species. Recent studies have empirically investigated the performance of species tree methods from multilocus datasets in a variety of organisms, including birds [3,33-36], insects [37,38], newts [39], plants [40], primates [41,42], rice [1], rodents [43], snakes [44], and yeast [4,16,36,45,46]. While some of these studies constructed highly-supported species trees, others did not, possibly due to high levels of genealogical discordance resulting from incomplete lineage sorting, hybridization, and ancient rapid radiations.

In one such study, [47] found that samples from a multilocus dataset of North American pines displayed widespread genealogical discordance. This pattern of incomplete lineage sorting is a common feature of long-lived shrubs and trees (e.g., [48-50]), and likely arises from factors such as large effective population sizes and long generation times [51]. Because gene tree discordance is needed for different algorithms to produce different estimates, high levels of gene tree discordance make North American pines an interesting group in which to compare species tree inference methods.

In this study, we take an empirical approach to the evaluation of species tree inference methods by examining the performance of 72 strategies for inferring species tree topologies using multilocus data from North American pines. Each “phylogenetic inference strategy” consists of three components: a method of constructing species trees from gene trees (e.g., consensus or concatenation), a gene tree inference method (e.g., maximum likelihood, maximum parsimony, or neighbor-joining), and an outgroup species. Our framework thus focuses on two-stage inference strategies that can be separated into gene tree inference and species tree inference steps, so that the effect of the choices of gene tree and species tree estimators can be directly evaluated. We examine ∼47 kilobases (kb) of sequence spanning 121 nuclear loci sequenced in 120 individuals from eight ingroup species of *Pinus* subsection *Strobus* (Table 1) and three outgroup species of *Pinus* subsection *Gerardianae*. With our empirical approach, unlike in simulation-based and theoretical evaluations, the true species tree is not known. It is still possible, however, to evaluate properties of species tree estimators without knowledge of a true tree, by comparing the features of species trees inferred by different methods. We apply techniques from multivariate statistical analysis to sets of inferred species trees to compare characteristics of species trees estimated by different strategies and to identify groups of strategies that behave similarly.

**Table 1:** **Ranges and morphological characteristics differentiating eight North American species of *Pinus *** **subsection *Strobus***

**Taxa**	**Common name**	**Range**	**Elevation (m)**	**Seed cone**	**Seeds**	**Dispersal**	**Related notes**
				**length (cm)**			
*P. albicaulis*^1^	Whitebark pine	Central British Columbia and Alberta south to central California; northern Rockies west to the Cascades and Sierra Nevadas	1300-3700	4-8	Wingless	Birds and rodents	Closed cone morphology where scales are opened through animal agency exclusively; timberline species.
*P. ayacahuite*^2^	Mexican white pine	Central Mexico to Honduras; sympatric with *P. chiapensis* at lower elevations	2300-3200	14-40	Winged	Wind	Southernmost species of the North American subsection *Strobus*; among the largest of Mexican pines.
*P. chiapensis*^2^	Chiapas pine	Veracruz, Mexico to northwestern Guatemala; sympatric with *P. ayacahuite* at upper elevations	260-2300	8-16	Winged	Wind	Formally considered as a disjunct population of *Pinus strobus* var. *chiapensis*.
*P. flexilis*^1^	Limber pine	Rocky Mountains and Intermountain Ranges from Canada south into the central US	1500-3600	7-15	Wingless	Birds and rodents	Often found at timberline; oldest trees date beyond 1600 years.
*P. lambertiana*^1^	Sugar pine	Oregon, California, Nevada, and isolated population in northern Baja California	330-3200	25-50	Winged	Wind	Largest species and longest seed cone of *Pinus*; unable to hybridize with any other North American pine.
*P. monticola*^1^	Western white pine	Southern British Columbia to south-central California; northern Rockies, Cascades, and Sierra Nevadas	0-3000	10-25	Winged	Wind	Found in moist, montane forests while most other western species are relegated to drier and more exposed sites.
*P. strobiformis*^1^	Southwestern white pine	Northern Mexico extending into central Arizona and New Mexico	1900-3500	15-25	Wingless	Birds and rodents	Range intergrades with *P. ayacahuite* to the south and *P. flexilis* to the north, and with these two species, forms a well-documented complex.
*P. strobus*^1^	Eastern white pine	Southern Canada south to Georgia; Newfoundland to western Ontario and Minnesota	0-1500	8-20	Winged	Wind	Only member of this group to occur in the eastern US and Canada; allopatric from all other taxa in subsection *Strobus*.

^1^[52].

^2^[53].

## Methods

### North American white pine dataset

A total of 120 individuals were sequenced in eight ingroup species of North American white pines from *Pinus* subsection *Strobus* (*Pinus albicaulis* Engelmann, *P. ayacahuite* Ehrenberg ex Schlechtendal, *P. chiapensis* (Martínez) Andresen, *P. flexilis* James, *P. lambertiana* Douglas, *P. monticola* Douglas ex D. Don, *P. strobiformis* Engelmann, and *P. strobus* L.) and three outgroup species from *Pinus* subsection *Gerardianae* (*P. bungeana* Zuccarini ex Endlicher, *P. gerardiana* Wallich ex D. Don, *P. squamata* X. W. Li), the identified sister lineage to *Pinus* subsection *Strobus*[47,54]. Sequencing was conducted on haploid templates generated from DNA extractions of seed megagametophyte tissue; as a single haploid sequence was generated for each individual at each locus, no phasing was necessary. Gene sequences were obtained from 245 putative nuclear loci chosen from among ∼7,500 loci recently resequenced for loblolly pine (*Pinus taeda* L., http://loblolly.ucdavis.edu/bipod/ftp/) using single pass, bidirectional Sanger sequencing of PCR products amplified from haploid megagametophyte tissue excised from seeds of each species. Further description of laboratory protocols appears in [55]. Sequence data were pre-processed and organized using PINESAP[56], a bioinformatics pipeline that combines PHRED[57], PHRAP[58], and MUSCLE[59,60] to call bases and align sequencing reads. Reported nucleotide sequences consisted only of A, C, G, T, missing, and gap information, with no other ambiguity codes used. After pre-processing, the data were manually assembled and aligned using CODONCODE (CodonCode Corporation, Dedham, MA). Bases were called using a minimum PHRED score [57,61] of 25 for aligned bases. All polymorphisms were visually validated. All alignments were further aligned to resequencing data from *P. taeda* (unpublished data) using the profile-profile option in MUSCLE[59,60]. These alignments are publicly available as part of the Dendrome project (http://loblolly.ucdavis.edu/bipod/ftp/). GenBank accession numbers for sequences in the study appear in Additional file 1: Table S1.

Of 245 loci sequenced initially, 37 were dropped from further consideration due to low overall quality of sequence reads. An additional 15 loci were discarded due to possible chloroplast or mitochondrial contamination, on the basis of BLAST analysis against pine organellar sequences in GENBANK[62]. Two loci were dropped due to sequence similarity to retroelement-like proteins, leaving 191 high-quality nuclear gene alignments. We then eliminated 70 loci for which at least one of the 11 species contained no data. This filter reduced the dataset to 121 loci, covering ∼47 kb of aligned sequence data.

Coding regions (*i.e.*, site annotations) could be confidently identified for 112 of the 121 loci by further analysis using TBLASTX against protein-coding genes in *Arabidopsis*, *Oryza*, *Picea*, and *Populus*. For these 112 loci, the gene for the highest-scoring TBLASTX hit, in combination with the expressed sequence tag from loblolly pine, was used to identify coding regions. Site annotations for each alignment were validated with BLASTP analysis of the amino acid sequences derived from the inferred coding intervals against the gene that was used to derive the site annotations. For the data from the 112 annotated loci, ∼62% represents exonic regions, ∼18% represents intronic regions, ∼1% is from 5’ UTRs, and ∼19% is from 3’ UTRs. Because 112 of the loci could be confidently identified as belonging to coding regions, with a substantial fraction of exonic sequence, the data likely contain a mixture of non-neutral and neutral regions.

### Overview of the analysis

**Figure 1:**
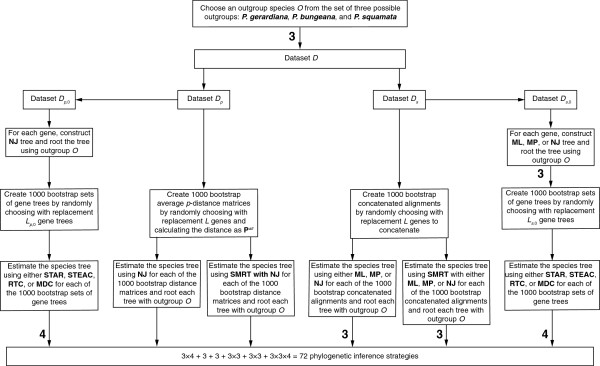
**Flow diagram representing the procedure in which we obtained results on the behavior of phylogenetic inference strategies.** A boldface number attached to a downward arrow indicates the number of phylogenetic inference strategies generated by the box immediately above the arrow. Absence of a number indicates a value of 1. The number of strategies for a particular path from the topmost box to the bottom layer is calculated as the product of the boldface numbers visited during the traversal of the path. The number of phylogenetic inference strategies analyzed is 72, the sum over all paths from the topmost box to boxes in the bottom layer.

The procedure for obtaining results for each of the 72 phylogenetic inference strategies (listed in Additional file 2: Table S1) appears in Figure 1. For a given strategy, we started from a dataset *D* with *L* loci. To generate distributions on the set of clades inferred by a given strategy, we used the bootstrap, creating bootstrap replicates by randomly choosing with replacement *B* sets of *L* loci. As many of the loci are coding and the eight pine species are closely related, we chose not to bootstrap across sites within a locus to ensure that bootstrapped alignments would contain reasonable levels of variation. Next, we applied a gene tree inference method to each bootstrap replicate dataset. Based on the set of inferred gene trees in a bootstrap replicate, we then applied a species tree construction method to estimate a species tree topology with one of the three outgroup species. For each phylogenetic inference strategy, we constructed *B*=1000 independent bootstrap datasets, thereby estimating 1000 species tree topologies. From these topologies, we created a list of clades, each with a corresponding count of its number of appearances in the 1000 bootstrap replicates. Clade lists were then analyzed to assess differences among the estimates produced by different strategies.

### Creating datasets

Our final set of 121 loci contains many loci that are highly conserved across multiple species. Because of the high level of conservation, for these loci, little information exists for identifying relationships among lineages. Thus, if methods for inferring gene trees were applied to certain loci, the resulting gene trees would be highly unresolved and would therefore provide little information to species tree construction methods. This issue motivates the construction of datasets that attempt to reduce the chance of inferring highly unresolved trees, and that provide phylogenetic inference strategies with the maximal amount of sequence data available.

We therefore analyzed four carefully selected subsets of the initial dataset (Table 2; Additional file 3). Two of these are datasets of multiple alignments that contain information on a single individual per species ($D_{s}$ and $D_{s,0}$). The other two contain information on multiple individuals per species ($D_{p}$ and $D_{p,0}$). These four datasets are constructed such that each possesses desirable properties for certain strategies in the collection of 72 phylogenetic inference strategies, providing the strategies with as much information as possible to infer resolved phylogenies. For example, because it is desirable for a pair of species to have nonzero distance, we require pairs of distinct species to be separated by at least one observed mutation. Furthermore, because it is desirable to minimize missing data, we choose individuals that yield minimal missing data in a multiple alignment. One of the two datasets with a single individual sampled per species is optimized for locus-by-locus gene tree inference ($D_{s,0}$), whereas the other is optimized for gene tree inference from a concatenated alignment ($D_{s}$). Similarly, one of the two datasets with multiple individuals sampled per species is optimized for locus-by-locus gene tree inference ($D_{p,0}$), whereas the other is optimized for gene tree inference using multiple loci simultaneously ($D_{p}$). The procedures used for constructing these datasets appear in Sections on “Datasets with one individual per species” and “Datasets with multiple individuals per species”.

**Table 2:** Datasets

**Dataset**	**Strategies that use the dataset**	**Number of strategies**	**Description**
$D_{s}$	Concatenation or SMRT with ML, MP, or NJ	18	Consists of all 121 loci, with a single individual sampled from each of 11 species at each locus.
$D_{s,0}$	STEAC, STAR, RTC, or MDC with ML, MP, or NJ	36	Subset of $D_{s}$ requiring that each locus has at least one sequence difference between each distinct pair of species, other than pairs from distinct outgroups.
$D_{p}$	Concatenation or SMRT with M	6	Consists of the full dataset , which contains all individuals and all loci.
$D_{p,0}$	STEAC, STAR, RTC, or MDC with M	12	Subset of $D_{p}$ requiring that each locus has at least one sequence difference between each distinct pair of species, other than pairs from distinct outgroups.

**Figure 2:**
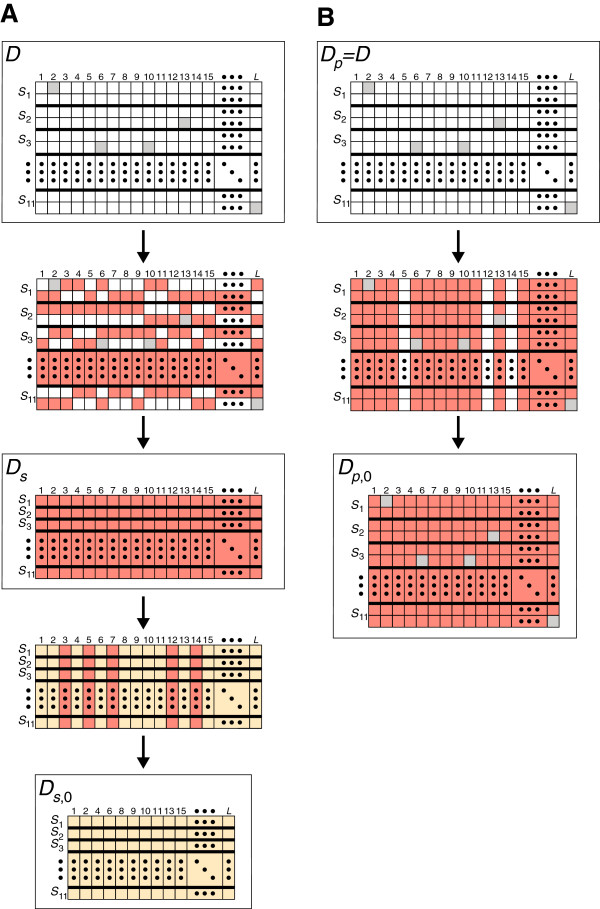
**Schematic for creating the four subsets**$D_{s}$**,**$D_{s,0}$**,**$D_{p}$**, and**$D_{p,0}$** from dataset****.** For the matrices of datasets , $D_{s}$, $D_{s,0}$, $D_{p}$, and $D_{p,0}$ (see Table 2), each row is an individual and each column is a locus. Thick black lines in these matrices separate the individuals in different species. Gray boxes indicate missing sequences. **(A)** At each locus, a single sequence from each species (indicated in red) is selected from dataset . These selected sequences are used to create $D_{s}$ such that there exists a single sequence sampled per species at each locus. Sequences from a subset of loci in $D_{s}$ (indicated in yellow) are used to create dataset $D_{s,0}$ such that each locus has at least one nucleotide difference between each distinct pair of species other than pairs from distinct outgroups. **(B)** Dataset $D_{p}$ is the full starting dataset . At each locus *ℓ*, a distance matrix is created according to eq. 2. Sequences from a subset of loci (indicated in red) in $D_{p}$ are used to create dataset $D_{p,0}$ such that each locus has a nonzero *p*-distance between each distinct pair of species other than pairs from distinct outgroups. Observe that the $D_{p,0}$ matrix includes loci 3 and 7, which are not included in the $D_{s,0}$ matrix. Loci 3 and 7 are included in $D_{p,0}$ but not in $D_{s,0}$ because in $D_{p,0}$, pairs of species contain at least one pair of individuals with different sequences, whereas in $D_{s,0}$, at least one pair of the 11 selected individuals have identical sequences. Therefore, the set of loci in $D_{p,0}$ is a superset of the set of loci in $D_{s,0}$, and the number of loci in $D_{p,0}$ is always greater than or equal to the number of loci in $D_{s,0}$.

Let *S*_*k*_, *k*=1,2,…,11, denote the set of individuals from pine species *k*, considering eight ingroup species (*S*_1_,*S*_2_,…,*S*_8_) and three outgroup species (*S*_9_,*S*_10_,*S*_11_). Denote the amount of overlapping non-gap non-missing sequence between a pair of individuals *x* and *y* by *n*_*xy*_ and denote the number of non-gap non-missing nucleotide differences between a pair of individuals *x* and *y* by *d*_*xy*_(0≤*d*_*xy*_≤*n*_*xy*_). Further, denote the final dataset of *L*=121 loci by $D=\{A_{1},A_{2},\ldots ,A_{L}\}$, where $A_{\ell }$ is the set of aligned sequences at locus *ℓ* for individuals from all 11 species. It is from dataset  that we create the four optimized datasets as summarized in Table 2 and Figure 2.

#### Datasets with one individual per species

The first dataset, $D_{s}$, consists of alignments with a single individual sampled per species at each locus (not necessarily the same individual across loci). That is, we generate a dataset of multiple alignments at each of *L* loci with only one individual per species, thereby creating multiple alignments of 11 individuals. This dataset is used by phylogenetic inference strategies that utilize the concatenation-based species tree construction methods with the maximum likelihood, maximum parsimony, and neighbor-joining gene tree inference methods (see “Inferring gene trees” and “Inferring species trees”). To create $D_{s}$, we choose the subset of 11 sequences $A_{\ell }^{s}$ at locus *ℓ* by first maximizing the total overlap sequence $n(A_{\ell }^{s})=\underset{x,y\in A_{\ell }^{s},x\neq y}{\sum }n_{\text{xy}}$ and then, if there is a tie for the overlap $n(A_{\ell }^{s})$, maximizing the total number of substitutions $d(A_{\ell }^{s})=\underset{x,y\in A_{\ell }^{s},x\neq y}{\sum }d_{\text{xy}}$. In other words, for any other set of aligned sequences $A_{\ell }^{R}\subseteq A_{\ell }$ at locus *ℓ* with a set *R* of only one individual sampled per species, the amount of overlapping non-gap non-missing sequence in $A_{\ell }^{R}$ is no larger than in $A_{\ell }^{s}$, *i.e.*, $n(A_{\ell }^{R})\leq n(A_{\ell }^{s})$. We note that the quantity *n*_*xy*_ represents a calculation only on a pair of individuals *x* and *y*, whereas $n(A_{\ell }^{s})$ considers all 112 pairs of individuals. Further, for any other set of aligned sequences $A_{\ell }^{R}\subseteq A_{\ell }$ at locus *ℓ* with a set *R* of only one individual sampled per species and $n(A_{\ell }^{R})=n(A_{\ell }^{s})$, the total number of pairwise sequence differences in $A_{\ell }^{R}$ is no larger than in $A_{\ell }^{s}$, *i.e.*, $d(A_{\ell }^{R})\leq d(A_{\ell }^{s})$. If multiple sets *R* of 11 individuals share the same values of *n* and *d*, then we choose the set of 11 individuals randomly among the tied sets. We choose the “optimal” set of 11 individuals at each locus in this way both to maximize the sequence contributions of individual loci to the inference of gene trees (maximizing *n*) and to maximize the potential for creating resolved gene trees (maximizing *d*).

The second dataset, $D_{s,0}$, is a subset of $D_{s}$ with *L*_*s*,0_≤*L* loci that consists of only those loci in $D_{s}$ for which there exists at least one nucleotide difference between each distinct pair of species (other than pairs of outgroup species). In other words, for any pair of individuals *x* and *y* with $x,y\in A_{\ell }^{s}$ and *x*≠*y*, *d*_*xy*_≥0, and *d*_*xy*_>0 if *x*, *y*, or both are from species 1 through 8. This condition of at least one nucleotide difference between species pairs assists in constructing gene trees that are bifurcating. Dataset $D_{s,0}$ is used by phylogenetic inference strategies that utilize consensus methods with maximum likelihood, maximum parsimony, and neighbor-joining (see “Inferring gene trees” and “Inferring species trees” for details).

#### Datasets with multiple individuals per species

The third dataset, $D_{p}$, is identical to our starting dataset . Thus, strategies that use $D_{p}$ consider all available sequences. Dataset $D_{p}$ is used by phylogenetic inference strategies that employ the concatenation-based species tree construction methods with the neighbor-joining gene tree inference method using multiple individuals (see “Inferring gene trees” and “Inferring species trees”).

Consider a dataset *D* of *L* loci sampled randomly with replacement from $D_{p}$. Define

(1) $$P_{\text{ij}}^{\text{all}}=\left\{\begin{array}{ll} 0 & ,i=j\\ \frac{\underset{A_{\ell }\in D}{\sum }\underset{x,y\in A_{\ell }}{\sum }d_{\text{xy}}1_{\left\{x\in S_{i},y\in S_{j}\right\}}}{\underset{A_{\ell }\in D}{\sum }\underset{x,y\in A_{\ell }}{\sum }n_{\text{xy}}1_{\left\{x\in S_{i},y\in S_{j}\right\}}} & ,i\neq j, \end{array}\right)$$

where the indicator random variable $1_{\left\{x\in S_{i},y\in S_{j}\right\}}$ equals 1 if *x*∈*S*_*i*_ and *y*∈*S*_*j*_ and 0 otherwise. The distance matrix defined by eq. 1 is used to estimate gene trees for all strategies applied to $D_{p}$. Given distinct species *S*_*i*_ and *S*_*j*_, $P_{\text{ij}}^{\text{all}}$ represents the *p*-distance (fraction of nucleotide differences; [63]) averaged over pairs of individuals, one from species *i* and the other from species *j*. Note that eq. 1 represents a weighted rather than unweighted average for the mean *p*-distance between species *i* and *j*. Although the distance is weighted, it is the same as a distance between pairs of species calculated on a concatenated alignment.

The fourth dataset, $D_{p,0}$, is a subset of $D_{p}$ with *L*_*p*,0_≤*L* loci. This subset consists of only those loci in $D_{p}$ for which there exists a pair of individuals in each distinct pair of species (other than pairs from distinct outgroups) with at least one nucleotide difference between them. Define

(2) $$P_{\text{ij}}^{\ell }=\left\{\begin{array}{ll} 0 & ,i=j\\ \frac{\underset{x,y\in A_{\ell }}{\sum }d_{\text{xy}}1_{\left\{x\in S_{i},y\in S_{j}\right\}}}{\underset{x,y\in A_{\ell }}{\sum }n_{\text{xy}}1_{\left\{x\in S_{i},y\in S_{j}\right\}}} & ,i\neq j, \end{array}\right)$$

where $1_{\left\{x\in S_{i},y\in S_{j}\right\}}$ is an indicator random variable that equals 1 if *x*∈*S*_*i*_ and *y*∈*S*_*j*_ and 0 otherwise. The numerator of $P_{\text{ij}}^{\ell }$ represents the number of pairwise sequence differences, summed over pairs of individuals, one from species *S*_*i*_ and the other from species *S*_*j*_, at locus *ℓ*. The denominator represents the sum across pairs of individuals, one from *S*_*i*_ and the other from *S*_*j*_, of the non-gap non-missing sequence shared between pairs of individuals at locus *ℓ*. To construct $D_{p,0}$, we create a subset of $D_{p}$ that consists only of those loci in $D_{p}$ for which the *p*-distance ($P_{\text{ij}}^{\ell }>0$) is nonzero between each distinct pair of species (excluding pairs from distinct outgroups). This dataset is utilized by phylogenetic inference strategies that employ consensus methods with gene trees inferred by neighbor-joining using multiple individuals (see “Inferring gene trees” and “Inferring species trees”). Similarly to dataset $D_{s,0}$, this condition of a nonzero *p*-distance between species pairs assists in constructing bifurcating gene trees. We note that the species tree estimation approach taken in this study neither requires pairs of individuals in the same species to have nonzero distances nor to have distances of zero. We only enforce that the distance calculated between pairs of species is nonzero.

### Inferring gene trees

For each of the four datasets $D_{s}$, $D_{s,0}$, $D_{p}$, and $D_{p,0}$, we inferred gene trees from bootstrap samples [63-65] that contain loci randomly sampled with replacement from the dataset. For strategies applied to datasets $D_{s}$ and $D_{s,0}$, we inferred gene trees from sequence alignments by applying either maximum likelihood (ML; [63], ch. 9) under a general time-reversible substitution model ([63], ch. 13), maximum parsimony (MP; [63], ch. 1), or neighbor-joining (NJ; [63], ch. 11) to a *p*-distance matrix calculated between pairs of alignments. For strategies applied to $D_{p}$ and $D_{p,0}$, we inferred gene trees by applying neighbor-joining to the **P**^*all*^ and **P**^*ℓ*^*p*-distance matrices, respectively. We term the method for inferring gene trees from the **P**^*all*^ and **P**^*ℓ*^*p*-distance matrices “neighbor-joining using multiple individuals” (M). Gene trees were inferred using PAUP^∗^[66]. Note that the estimation of gene trees on the scale explored in this study would be computationally intensive on the full set of sampled individuals; thus, we do not consider gene tree inference directly from alignments with multiple lineages sampled within species, and when exploring multiple lineages (as in M), we do so only with distance matrices between pairs of species rather than pairs of lineages.

### Inferring species trees

We view as a species tree inference method any method that outputs a species tree estimate. The six species tree inference methods in this study are Concatenation [16,67], SuperMatrix Rooted Triple (SMRT; [22]), STEAC [21], STAR [21], Rooted Triple Consensus (RTC; [68]), and Minimize Deep Coalescences (MDC; [69,70]). Concatenation and SMRT are concatenation-based, and STEAC, STAR, RTC, and MDC are consensus methods. Because we have adopted a unified two-stage framework for phylogenetic inference strategies in which gene trees are first inferred by one approach and species trees are then inferred from gene trees by a second approach, we did not investigate single-stage approaches such as BEST [18,19], and *BEAST [20] that bypass gene tree inference or that perform gene tree inference simultaneously with species tree inference. Our analysis pipeline explores the performance of two-stage inference strategies when the roles of gene tree and species tree inference are separated, and it therefore requires that strategies estimate species trees from inferred gene trees and that they permit different gene tree inference methods to provide input to a given species tree method. The six species tree methods investigated in this article satisfy both of these conditions, whereas species tree methods such as BUCKy [17], BEST [18,19], and *BEAST [20] do not. Further, the methods we have selected are well-suited to a computationally intensive bootstrap approach included in our pipeline for generating distributions of species tree topologies, and the more computationally intensive of the single-stage methods would not be easily accommodated within this framework. Given the large number of two-stage methods available, it would not be possible to be comprehensive; we have thus chosen a limited number of methods that represent a range of underlying principles. Our choice of methods permits a diverse set of criteria for estimating species trees to be evaluated, and the conceptual differences in the underlying methods enable some differentiation in behavior across methods.

Consider a set of *L* loci (multiple alignments) with *m* ingroup and one outgroup species. Concatenation methods concatenate the *L* alignments to create a single “super locus” consisting of an alignment of the *m*+1 species across *L* loci. From this alignment, a gene tree is inferred by either maximum likelihood, maximum parsimony, or neighbor-joining—note that the definition of Concatenation does not require that gene trees be estimated using any specific method—and is then taken as the species tree estimate. Similarly, SMRT creates a concatenated alignment of the *m*+1 species from a set of *L* alignments. However, SMRT then constructs from this concatenated alignment all m3 concatenated alignments of three ingroup species and an outgroup species. Rooted three-taxon gene trees are then inferred from each of the m3 concatenated alignments. A supertree algorithm is then applied to the set of rooted three-taxon gene trees to estimate an *m*-taxon species tree topology. This study uses the Modified Mincut supertree algorithm implemented in the program SUPERTREE[71] to construct a species tree from rooted three-taxon gene trees.

Consider a set of (*m*+1)-taxon gene trees (*m* ingroup and one outgroup species) inferred at each of *L* loci. STEAC estimates a species tree topology by using estimated mean coalescence times. For distinct species *S*_*i*_ and *S*_*j*_, the mean coalescence time is computed as the estimated coalescence time for *S*_*i*_ and *S*_*j*_, averaged over all *L* gene trees. This resulting mean is placed into a matrix of distances between species, to which neighbor-joining is applied to estimate the species tree topology (using the R PHYBASE package).

STAR estimates a species tree topology by using average coalescence ranks. It assumes that the rank of the root of a gene tree is equal to the number of species in the tree (*m* + 1 in our case). An internal node of a gene tree is then assigned one less than the rank of its immediate ancestor. For distinct species *S*_*i*_ and *S*_*j*_, the average coalescence rank is computed as the rank of the node that connects *S*_*i*_ and *S*_*j*_, averaged over all *L* gene trees. Similarly to STEAC, these average coalescence ranks specify a matrix of distances between species pairs. Neighbor-joining is applied to the matrix to estimate the species tree topology using PHYBASE.

RTC estimates a species tree from rooted three-taxon tree topologies. At each locus *ℓ*, *ℓ*=1,2,…,*L*, RTC finds the set of m3 rooted tree topologies of three ingroup and one outgroup species that are displayed by the inferred gene tree at locus *ℓ*. RTC then applies quartet puzzling [72] to the m3L topologies to estimate the species tree topology (using the program TRIPLEC).

A coalescence event between a pair of lineages is considered “deep” if the coalescence does not occur in the first population in which the pair of lineages is capable of coalescing. Given a gene tree, the number of deep coalescences on a species tree is defined as the total number of “extra lineages”, summed across branches of the species tree topology, that is needed to fit the gene tree within the species tree topology. Here, the number of extra lineages for a branch is one fewer than the number of lineages that survive to the ancestral node of the branch; if incomplete lineage sorting does not occur, then only one lineage persists from a branch to a more ancestral branch, and there are no extra lineages. For a set of *L* gene trees, the number of deep coalescences for a species tree is the total number of deep coalescences for the species tree given a gene tree, summed across the *L* gene trees. MDC estimates a species tree topology by minimizing the number of deep coalescences. That is, MDC finds a species tree topology for which the number of deep coalescences that will fit the set of *L* gene trees within the species tree topology is minimal. This study utilizes the MDC implementation in PHYLONET[70].

### Multivariate analysis

We aim to determine which of the 72 phylogenetic inference strategies perform similarly, and we use multivariate analyses to define clusters of strategies that provide similar species tree estimates. Consider a 72×145-dimensional data matrix **S** in which rows represent strategies and columns represent 145 observed clades, among the ∑k=28-18k=246 possible non-trivial clades (*i.e.*, clades that contain more than one species and fewer than all analyzed species) of eight species. Entry **S**_*ij*_ in column *i* and row *j* of **S** is the number of times that strategy *i* infers clade *j* in 1000 bootstrap replicates across loci.

Principal components analysis (PCA) was applied to **S** to create a 72×2-dimensional matrix **V**, with rows representing strategies and the first and second columns representing the first and second principal components, respectively. Plotting strategies onto the space defined by these principal components yields a two-dimensional spatial “map” of phylogenetic inference strategies. We similarly applied multidimensional scaling (MDS) to a distance matrix for all 722 pairs of strategies, computing pairwise distance as the mean Robinson-Foulds distance [73] across all 10^6^ pairs of bootstrap trees, and extracting the first two components. We calculated the Robinson-Foulds distance using TREEDIST in PHYLIP.

To compare spatial maps of phylogenetic inference strategies, we used Procrustes analysis [74-76]. In particular, we compared the spatial distribution of a subset of 72-*r* strategies when analyzed alone to the spatial distribution for all strategies. The comparison enabled us to quantify the influence that a set of *r* strategies with a particular feature (*i.e.*, species tree construction method, gene tree inference method, or outgroup species) has on the full spatial distribution. Consider a proper subset *Σ*={*σ*_1_,*σ*_2_,…,*σ*_72-*r*_} of the full set of strategies. Consider a (72-*r*)×145-dimensional data matrix **S**_*Σ*_ in which rows represent the strategies in set *Σ* and columns represent observed clades. **S**_*Σ*_ is a submatrix of **S**, in which the rows corresponding to strategies in *Σ* are selected from **S**. Consider a (72-*r*)×2 target matrix **X** and a (72-*r*)×2 comparison matrix **Y**. **X** is matrix **V** restricted to the set of strategies *Σ*. **Y** represents the first two principal components in the PCA applied to matrix **S**_*Σ*_. Now consider a (72-*r*)×2 matrix **Z**=*b***YT**+**C**, where *b* is a scaling factor, **T** is a 2×2 matrix that rotates and reflects **Y**, and **C** is a (72-*r*)×2 matrix that has constant columns and that is used to translate the matrix. Procrustes analysis seeks to find *b*, **T**, and **C** to minimize the sum of squared differences between **X** and some (72-*r*)×2 matrix **Z**^⋆^=*b***YT**+**C**. That is, **Z**^⋆^ is formally defined as $Z^{⋆}={\text{argmin}}_{Z}\{\overset{72-r}{\underset{i=1}{\sum }}\overset{2}{\underset{j=1}{\sum }}{\left(X_{\text{ij}}-Z_{\text{ij}}\right)}^{2}\}$. The dissimilarity measure between **X** and **Z**^⋆^ is computed as

(3) $$\frac{\overset{72-r}{\underset{i=1}{\sum }}\overset{2}{\underset{j=1}{\sum }}{\left(X_{\text{ij}}-Z_{\text{ij}}^{⋆}\right)}^{2}}{\overset{72-r}{\underset{i=1}{\sum }}\overset{2}{\underset{j=1}{\sum }}{\left(X_{\text{ij}}-\mu_{j}\right)}^{2}},$$

where $\mu_{j}=\frac{1}{72-r}\overset{72-r}{\underset{i=1}{\sum }}X_{\text{ij}}$ is the *j*th entry of the centroid of **X**. This measure takes the sum of squared differences between points on the spatial maps defined by **X** and **Z**^⋆^ and normalizes it by the sum of squared differences between the points on the spatial map defined by **X** and their centroid.

Define a cluster as a set of strategies and let the centroid of a cluster be the location in the 145-dimensional space of clades whose coordinates are the means of those of all strategies in the cluster. Hierarchical clustering was performed by first creating a matrix of Euclidean distances between all 722 pairs of 145-dimensional vectors represented by the matrix **S**. Define the within-cluster sum of squared Euclidean distance as the squared Euclidean distance between a point in a cluster and the cluster centroid, summed over all points in the cluster. From the 72×72-dimensional matrix of Euclidean distances between strategies, a dendrogram relating the strategies was constructed using the Ward algorithm [77], which iteratively merges clusters until all points are contained within a single cluster. For a given iteration, two clusters are merged if their merged cluster has a smaller within-cluster sum of squared Euclidean distances than any other potential merged cluster. The nesting of clusters created by the algorithm defines the dendrogram.

We performed *K*-means clustering on the 72 145-dimensional vectors, using *K* clusters, *K*=2,3,…,9. Given *K*, strategies were separated into *K* clusters on the basis of the squared Euclidean distance between all pairs of the strategies in a 145-dimensional space. We ran 10^4^ replicates with random starting locations. Each replicate yielded a total within-cluster sum of squared distances for the set of *K* clusters, representing the within-cluster sum of squared distances between points in a cluster and the cluster centroid, summed over all *K* clusters. We then chose the set of cluster assignments that had the minimum total within-cluster sum of squared distances, where the minimum was taken over all 10^4^ replicate starting locations.

To compute the Pearson correlation coefficient between a pair of strategies, we only used points in the 145-dimensional vector that were nonzero in both strategies being compared.

## Results

We accounted for the variable outcomes of individual phylogenetic inference strategies by applying the strategies to bootstrap datasets instead of their respective full datasets. Our analysis identified 145 distinct clades observed in the set of 72 phylogenetic inference strategies, among 246 possible non-trivial clades on eight species, across 1000 bootstrap replicates for each strategy. From these clades, we created a 72×145 matrix **S** in which each row is a strategy and each column is a clade. The value of **S**_*ij*_, the cell in row *i* and column *j*, is the number of times among the 1000 bootstrap replicates that strategy *i* inferred a species tree with clade *j*. This summarized dataset **S** of clade counts was used for all further analyses.

### Clade size

We first investigated the level of balance [78-81] in the tree topologies inferred by each phylogenetic inference strategy. The distribution of clade sizes (number of taxa within a clade) provides a basis for measuring tree topological balance. Topologies with numerous small clades tend to be more balanced than topologies with large clades. For example, consider the topologies *T*_*bal*_=(((*AB*)(*CD*))((*EF*)(*GH*))) and *T*_*unbal*_=(((((((*AB*)*C*)*D*)*E*)*F*)*G*)*H*). Topology *T*_*bal*_ is the most balanced eight-taxon topology whereas *T*_*unbal*_ is the most unbalanced eight-taxon topology. Considering non-trivial clades, *T*_*bal*_ has four clades of size two and two clades of size four. *T*_*unbal*_ has one clade each of size two, three, four, five, six, and seven. Thus, the clades of *T*_*bal*_ are smaller than those of *T*_*unbal*_. The mean clade size for *T*_*bal*_ is ∼2.67 and the mean clade size for *T*_*unbal*_ is 4.5.

**Figure 3:**
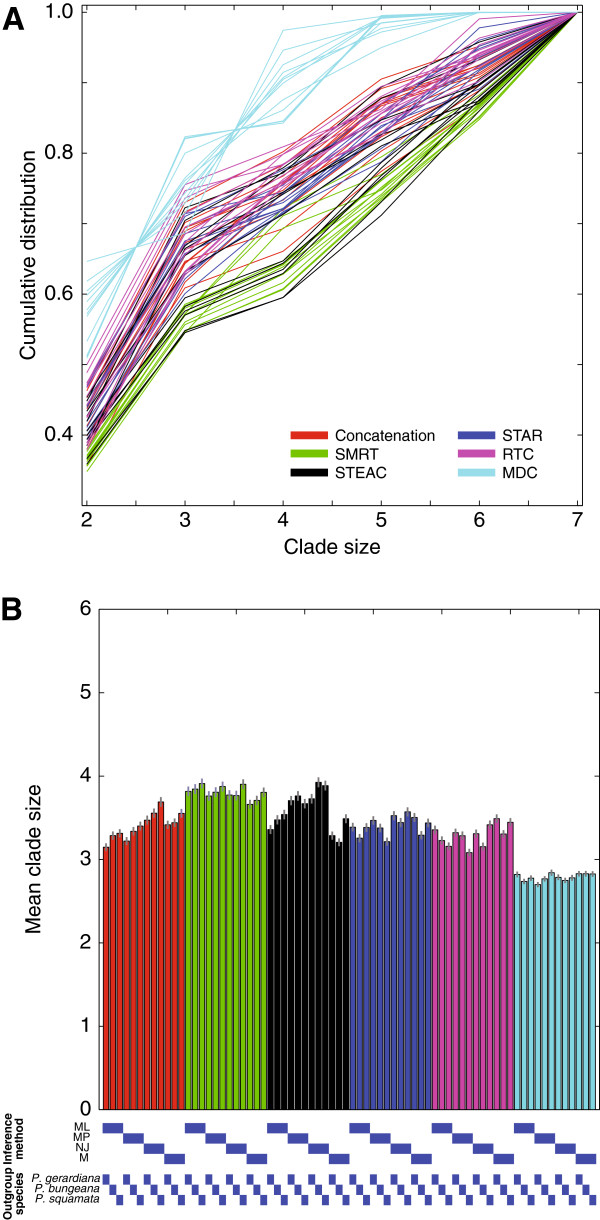
**Distribution of clade size for phylogenetic inference strategies.(A)** Cumulative distribution of clade sizes. Each bar represents a strategy, of which there are 12 per color. **(B)** Mean clade size for phylogenetic inference strategies, calculated as the mean size of all clades inferred across 1000 bootstrap replicates. Vertical lines centered at the top of vertical bars represent standard errors of mean clade sizes.

Figure 3A displays the cumulative distribution of clade sizes for each of the 72 phylogenetic inference strategies, considering all 1000 bootstrap replicate species trees for each strategy. This cumulative distribution increases most quickly for strategies based on MDC, for which most of the distribution is located in clades of size two. By contrast, it increases most slowly for strategies based on SMRT and STEAC, for which much of the distribution is located in clades of size six and seven. Figure 3B displays a bar graph of the mean clade size for each of the 72 phylogenetic inference strategies. This graph shows that among all six species tree construction methods, the 12 MDC strategies have the smallest mean clade size as well as the smallest variance in mean clade size across the 12 combinations of outgroup and gene tree inference method. In contrast, SMRT and STEAC in general have the largest mean clade size. However, all 12 SMRT strategies infer trees with large mean clade size, whereas the mean clade size of STEAC varies across the 12 combinations of outgroup and gene tree inference method. Interestingly, the mean clade size averaged over all 12 strategies based on MDC is ∼2.79, a value that is close to the mean clade size for *T*_*bal*_ of ∼2.67.

### Clustering of strategies

**Figure 4:**
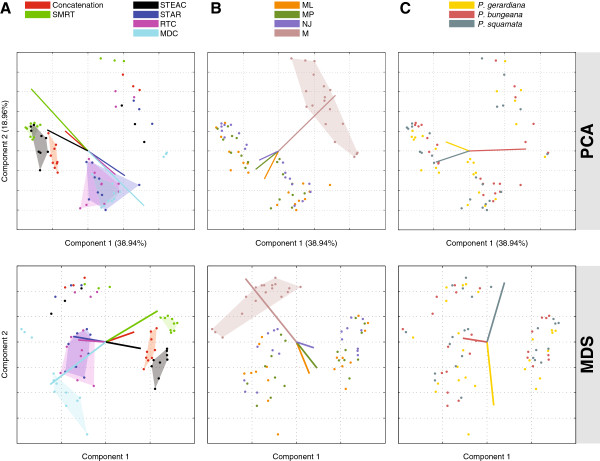
**Principal components analysis (PCA) and multidimensional scaling (MDS) of phylogenetic inference strategies.** PCA was applied to 72 phylogenetic inference strategies, in which each strategy is viewed as a point in a 145-dimensional space of clades. MDS was applied to a distance matrix between all pairs of strategies, where the distance between a pair of strategies was the mean Robinson-Foulds distance between all pairs of bootstrap trees from one strategy and another strategy (*i.e.*, mean of 10^6^ comparisons). The plots show the first and second components. On a given row, each plot represents the same 72 points in the space of components 1 and 2; the three plots are colored differently to highlight different features. **(A)** Colors represent different methods for constructing species trees. **(B)** Colors represent different gene tree inference methods. **(C)** Colors represent different outgroups. The points on each graph represent different combinations of the three factors that form phylogenetic inference strategies. Each line in part **A** represents the resultant vector (scaled by a constant to lie within the span of the 72 points) for all 12 points of a certain method for constructing species trees. Each line in part **B** represents the resultant vector for all 18 points of a gene tree inference method (scaled by a constant). Each line in part **C** represents the resultant vector for all 24 points of an outgroup (scaled by a constant). The scaling constants in parts **A**, **B**, and **C** are distinct. Each of the shaded regions in parts **A** and **B** is a convex hull of the points from a particular method.

We next used PCA, MDS, hierarchical clustering, *K*-means clustering, and correlation analysis on the matrix of clades **S** to identify phylogenetic inference strategies that perform similarly. Figure 4 displays plots of the first two principal components, which account for 38.94% and 18.96% of the variation across strategies, respectively. Figure 4A shows that separate clusters are formed by strategies that are based on Concatenation, SMRT, and STEAC, and that strategies based on STAR, RTC, and MDC form a cluster together. Further, a larger cluster is formed by strategies that are based on Concatenation, SMRT, and STEAC, and another larger cluster is formed by strategies that are based on STAR, RTC, and MDC. These larger clusters have a simple interpretation in that one of the larger clusters contains topologically-based strategies (STAR, RTC, and MDC) and the other contains strategies that are not strictly topologically-based (Concatenation, SMRT, and STEAC). Strategies are classified as topologically-based if they only use information on tree topologies to construct a species tree. In contrast, strategies are classified as not strictly topologically-based if they use information other than the gene tree topologies, such as sequence or branch length information, to construct a species tree. Relabeling the points in Figure 4A according to gene tree inference method, Figure 4B shows that strategies that are based on M (*i.e*., multiple individuals) form a cluster, and that there are no separate clusters for strategies that are based on ML, MP, or NJ. Figure 4C, which labels points according to outgroup, shows that no strategies separate into clusters based on the choice of outgroup. When we apply MDS to Robinson-Foulds distances between the sets of bootstrap replicate trees produced by pairs of strategies (Figure 4), we obtain similar observations of the clusters of strategies, detecting an important role for M and for the difference between topologically-based and non-topologically-based strategies, and no strong signal for the outgroup choice.

From Figure 4, we can see that much of the variation across the 72 phylogenetic inference strategies, as explained by PCA and MDS, is caused by M. Strategies based on M are more similar in clade outcomes to other strategies based on M than they are to other strategies that are not based on M. The magnitude of this effect can be quantified using Procrustes analysis, which demonstrates that M has a large influence on the spatial relationship among all other phylogenetic inference strategies (Additional file 2: Figure S1).

**Figure 5:**
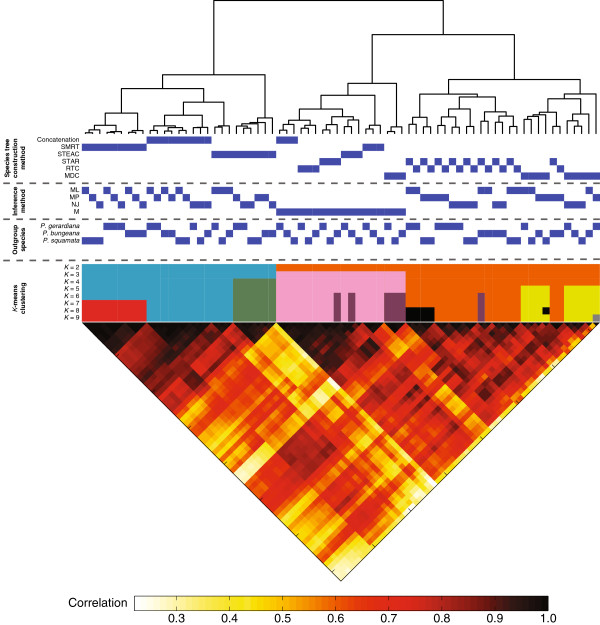
**Cluster and correlation analysis of phylogenetic inference strategies.** Each leaf of the dendrogram corresponds to a different phylogenetic inference strategy for obtaining the rooted phylogeny of eight ingroup pine species. Blue squares directly below the dendrogram indicate the features used to construct a rooted phylogeny for the eight pine species. The first six rows below the dendrogram represent different species tree construction methods. The next four rows below the dendrogram represent gene tree inference methods. The following three rows below the dendrogram represent the outgroup species. The dendrogram was constructed by hierarchical clustering using the Ward algorithm [77] applied to a matrix of Euclidean distances between all 722 pairs of 145-dimensional vectors (each dimension representing a distinct clade). The remaining nine rows below the outgroups show the results of *K*-means clustering applied to the 72 145-dimensional vectors with *K* clusters, *K*=2,3,…,9. Below the cluster analysis is a heat map of the correlation coefficients between all 722 pairs of phylogenetic inference strategies. An entry in the heat map represents the Pearson correlation coefficient between a pair of strategies by only using points in the 145-dimensional vector that were nonzero in both strategies being compared.

Figure 5 shows the results of our cluster and correlation analyses. The main clusters formed by phylogenetic inference strategies involve strategies based on the species tree construction methods Concatenation, SMRT, STEAC, and MDC or the gene tree inference method M (Figure 5). The clusters of strategies formed by *K*-means and the large groupings of strategies formed by hierarchical clustering are quite similar. Additionally, the correlation coefficient between clade vectors inferred by pairs of phylogenetic inference strategies is generally higher for pairs of strategies that are placed into the same cluster by either *K*-means or hierarchical clustering than for pairs of strategies that are not placed into the same cluster (Figure 5).

Interestingly, the clustering of strategies by PCA and MDS in Figure 4 matches well with the groupings observed in Figure 5, which is likely driven by similar signals. In Figure 5, three large clusters are represented by the subtree to the left of the root of the dendrogram (*i.e.*, the blue color in the *K*-means clustering with *K*=3) and by two subtrees to the right of the root (*i.e.*, the pink and orange colors at *K*=3). The two subtrees to the right of the root (or pink and orange clusters defined by *K*-means clustering) involve strategies that are based on M (pink *K*-means cluster or left subtree on the right of the root of the dendrogram) or strategies that are based on species tree construction methods that are topologically-based (orange *K*-means cluster or right subtree on the right of the root of the dendrogram). That is, strategies that correspond to the orange cluster are based on either STAR, RTC, or MDC. In contrast, the subtree to the left of the root (or the blue cluster defined by *K*-means clustering) contains only strategies that use species tree construction methods that are not strictly topologically-based (*i.e.*, Concatenation, SMRT, or STEAC).

From Figures 4 and 5, we find that phylogenetic inference strategies form three basic clusters: a cluster that involves strategies that are based on M, a cluster that involves strategies that are topologically-based, and a cluster that involves strategies that are not strictly topologically-based.

### Clade flow

**Figure 6:**
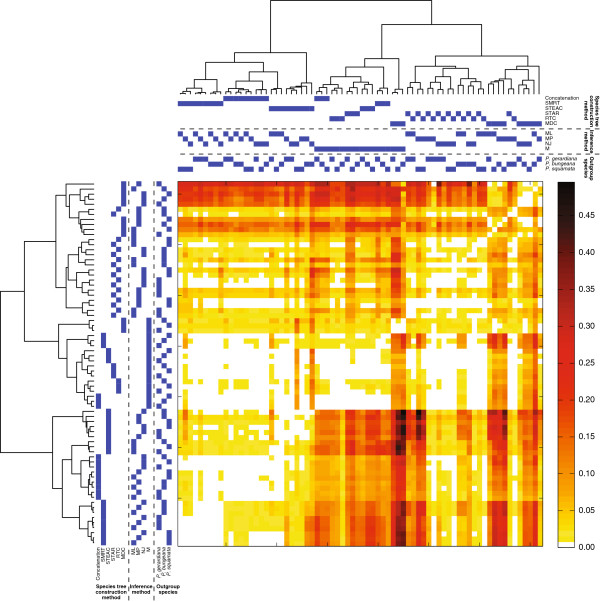
**Heat map representing the “flow” of clades between phylogenetic inference strategies.** We use clade flow to measure the proportion of clades inferred by one strategy that were not inferred by a different strategy. Phylogenetic inference strategies are ordered using the dendrogram. The cell at row *i* and column *j* represents the fraction of clades inferred by strategy *i* that were not inferred by strategy *j*. By definition, the heat map is asymmetric. Darker colors indicate lower levels of “flow” from a row to a column.

Following the “haplotype flow” computations of [82], we can view “clade flow” as a proportion of clades inferred by one phylogenetic inference strategy that are also inferred by another strategy. Figure 6 displays a heat map that represents a form of clade flow, where the cell at row *i* and column *j* in the heat map represents the fraction of clades inferred by strategy *i* that were not inferred by strategy *j*. By definition, the heat map is not symmetric. As can be seen from the mostly white and yellow boxes for rows corresponding to strategies based on M, these strategies tend to infer clades that are supported by other strategies. That is, if a species tree topology is inferred by a strategy that is based on M, then clades displayed by that topology will often also be present on species tree topologies inferred by other strategies. In Additional file 2: Figure S1, strategies based on M contribute to the most variation across strategies. A possible explanation for this observation is that the flow of clades is largely unidirectional. That is, if a strategy is based on M, then clades that are inferred by that strategy also tend to be supported by other strategies; however, if a strategy not based on M infers a clade, then that clade is not often supported by strategies based on M. Because clades inferred by strategies based on M also tend to be supported by other strategies, it follows that strategies based on M tend to infer clades that are also supported by other strategies based on M. This sharing of clades among strategies based on M causes those strategies to be more similar to each other than they are to strategies not based on M. In contrast to the results for M, as can be seen from the mostly dark boxes in rows for strategies based on MDC, strategies based on MDC tend to infer clades that are not supported by other strategies (especially when compared with strategies based on M).

Similarly to the behavior of MDC, strategies that are based on Concatenation, SMRT, and STEAC together with ML, MP, or NJ share more clades with other such strategies (mostly white and yellow boxes) than with the remaining strategies (mostly dark boxes). In contrast, as was observed with M, strategies based on STAR and RTC together with ML, MP, or NJ share similar numbers of clades among other such strategies as with the remaining strategies (mostly yellow boxes). These results suggest that strategies that are topologically-based (*i.e.*, STAR and RTC) tend to infer clades that are also supported both by other topologically-based strategies and by strategies that are not strictly topologically-based, whereas strategies that are not strictly topologically-based (*i.e.*, Concatenation, SMRT, and STEAC) tend to infer clades that are not supported by strategies that are strictly topologically-based (*i.e.*, STAR, RTC, and MDC).

### Representative topologies

**Figure 7:**
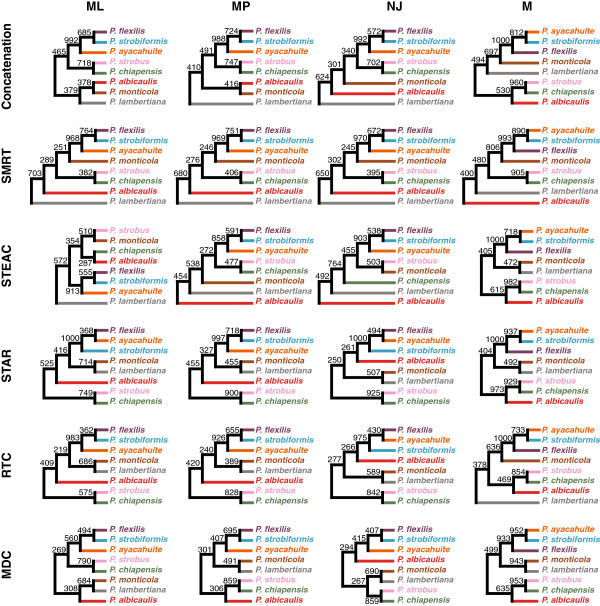
**Rooted consensus trees of phylogenetic inference strategies averaged over outgroups.** For a given subset of the 72 phylogenetic inference strategies considered, the bootstrap support for each of the clades that appeared in at least one tree was averaged over the set of strategies to create a set of counts for each of the clades. Greedy consensus trees [12] were then created using the clade counts in the set. Each clade count in the set has a maximum value of 1000, because each element of the set is an average over values that each have a maximum of 1000. Each consensus tree is the greedy consensus tree based on clade counts averaged over outgroup species. These trees disregard branch-length information.

**Figure 8:**
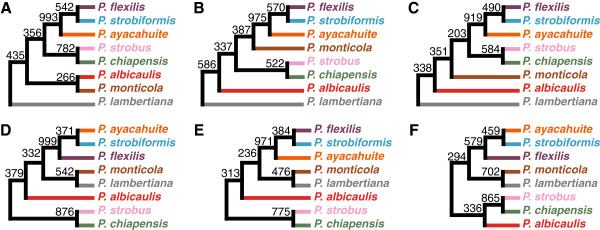
**Rooted consensus trees of phylogenetic inference strategies averaged over outgroups and gene tree inference methods.** For a given subset of the 72 phylogenetic inference strategies considered in this article, the bootstrap support for each of the clades that appeared in at least one tree was averaged over the set of strategies to create a set of counts for each of the clades. Greedy consensus trees [12] were then created using the clade counts in the set. Each clade count in the set has a maximum value of 1000, because each element of the set is an average over values that each have a maximum of 1000. These trees disregard branch-length information. **(A)** Trees constructed using the 12 strategies that utilize Concatenation; **(B)** SMRT; **(C)** STEAC; **(D)** STAR; **(E)** RTC; **(F)** MDC.

**Figure 9:**
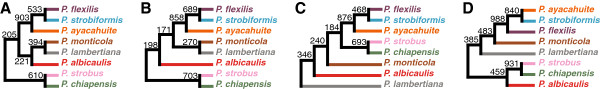
**Rooted consensus trees of phylogenetic inference strategies averaged over outgroups and species tree construction methods.** For a given subset of the 72 phylogenetic inference strategies considered in this article, the bootstrap support for each of the clades that appeared in at least one tree was averaged over the set of strategies to create a set of counts for each of the clades. Greedy consensus trees [12] were then created using the clade counts in the set. Each clade count in the set has a maximum value of 1000, because each element of the set is an average over values that each have a maximum of 1000. These trees disregard branch-length information. **(A)** Trees constructed using the 18 strategies that utilize ML; **(B)** MP; **(C)** NJ; **(D)** M.

We next wanted to use a set of representative species tree topologies to highlight similarities and differences in topologies constructed by various strategies. Topologies were estimated using the Greedy Consensus algorithm [12] applied to clade counts. Because our previous results (Figures 4-5) indicate that the choice of outgroup species does not strongly influence the overall inferred topologies, it is sensible to average across outgroups. Therefore, we first present topologies for each of the 24 species tree–gene tree inference method pairs constructed from clade counts that were averaged over the three outgroups (Figure 7). Next, to obtain a clearer picture of the types of topologies that are inferred by the six species tree inference methods, we present topologies for each of the six species tree inference methods, constructed from clade counts that were averaged over gene tree inference methods and outgroup species (Figure 8). Finally, to assess the influence that various gene tree inference methods have on the overall inferred species tree topology, we present topologies for each of the four gene tree inference methods, constructed from clade counts that were averaged over species tree inference methods and outgroup species (Figure 9).

Figure 7 displays 24 topologies with clade support values for each combination of a species tree construction method and a gene tree inference method. The clade {*P. chiapensis*, *P. strobus*} is generally highly supported, appearing for 22 of 24 strategies, with support ranging from 382 to 982 among 1000 bootstrap replicates. The smallest support values for {*P. chiapensis*, *P. strobus*} occur in strategies that use SMRT with ML, MP, and NJ, producing support values of 382, 406, and 395, respectively. The largest support values for this clade occur in strategies that use M, with values ranging from 824 to 982. Further, although strategies based on SMRT with ML, MP, and NJ yield lower support values than other strategies, when SMRT is combined with M, the support for {*P. chiapensis*, *P. strobus*} is 905. In addition, although two of the strategies based on STEAC do not support {*P. chiapensis*, *P. strobus*}, when STEAC is combined with M, the support for the clade is 982. Another clade that is highly supported is {*P. ayacahuite*, *P. flexilis*, *P. strobiformis*}. This clade is observed across all strategies, with support among non-MDC strategies out of 1000 bootstrap replicates ranging from 858 to 1000. Strategies that use MDC with ML, MP, and NJ yield support values for {*P. ayacahuite*, *P. flexilis*, *P. strobiformis*} of 560, 407, and 415, respectively. However, using MDC with M yields a support value of 933 for {*P. ayacahuite*, *P. flexilis*, *P. strobiformis*}. Across the 24 trees, the topological positions of *P. albicaulis*, *P. lambertiana*, and *P. monticola* are variable and are generally poorly supported. Each of these species is found in a variety of positions across all trees.

Figure 8 displays six topologies with clade support values for each species tree construction method. Similarly to Figure 7, the clade {*P. chiapensis*, *P. strobus*} is generally highly supported across all six species tree construction methods, with support ranging from 522 to 876 among 1000 bootstrap replicates. Also, as in Figure 7, the clade {*P. ayacahuite*, *P. flexilis*, *P. strobiformis*} is highly supported across all six species tree construction methods, with support ranging from 579 to 999 among 1000 bootstrap replicates. From these topologies, we can also observe that in agreement with the clade size distribution, strategies based on Concatenation, SMRT, and STEAC tend to produce more unbalanced trees than strategies based on STAR, RTC, and MDC (Figure 3). Strategies based on Concatenation, SMRT, and STEAC support topologies in which *P. lambertiana* is on the opposite side of the root from the other seven species. In contrast, strategies based on STAR, RTC, and MDC place *P. monticola* and *P. lambertiana* as sister species. These results support the observations from Figures 4, 5, and 6 that strategies based on species tree construction methods that are topologically-based behave differently from strategies that are not strictly topologically-based.

Figure 9 displays four topologies with clade support values, considering each gene tree inference method and combining species tree construction methods for each gene tree inference method. As in Figures 7 and 8, the clades {*P. chiapensis*, *P. strobus*} and {*P. ayacahuite*, *P. flexilis*, *P. strobiformis*} are generally highly supported across all four gene tree inference methods, with supports among 1000 bootstrap replicates respectively ranging from 610 to 931 and from 858 to 988.

## Discussion

In this article, we have empirically evaluated strategies for inferring species tree topologies from multilocus sequence data. We have found that MDC tends to infer balanced topologies, whereas SMRT and STEAC tend to infer more unbalanced topologies. This bias toward balanced topologies exhibited by MDC is a consequence of the nature of the criterion that MDC uses to construct species trees, reflecting a theoretical finding that species trees with more balance have lower deep coalescence costs [83].

The strategies that we have examined fall into three classes in terms of the species tree inferences they produce: strategies applied only to datasets including all available sequenced individuals (*i.e.*, M), topologically-based strategies (*i.e.*, STAR, RTC and MDC), and strategies that are not strictly topologically-based (*i.e.*, Concatenation, SMRT, and STEAC). While it is not unexpected that some approaches would behave similarly, it is surprising that strategies did not cluster based on the dataset or approach used (e.g., consensus or concatenation). Instead, strategies that take quite different species tree construction approaches (e.g., consensus-based STEAC and concatenation-based Concatenation and SMRT) form a cluster. Topologically-based strategies tend to infer clades that are supported by other strategies, whereas strategies that are not strictly topologically-based tend to infer clades that are not always well-supported by other strategies. For example, clades inferred from strategies that are not strictly topologically-based tend not to appear on trees that were inferred from strategies that are topologically-based. A possible reason for this observation could be a lack of phylogenetic signal to properly infer branch lengths; that is, if a phylogenetic inference strategy is not strictly topologically-based, then sequences with little phylogenetic signal (e.g., due to low substitution rate or short length) can strongly influence the species tree inferred by that strategy. Because STEAC uses branch-length information to infer a species tree topology, sequences with little signal can reduce its performance relative to topologically-based methods such as STAR [15].

Although our main goal has been to use North American pines to investigate relationships among phylogenetic inference strategies, our results also provide some information about the phylogenetic placement of the pine species in the study. This analysis is the first multilocus study to provide substantial confidence for a sister relationship of *P. chiapensis* and *P. strobus*. *Pinus chiapensis* is a threatened species of Mexico and Guatemala whose phylogenetic affinity has been uncertain. Morphological and molecular evidence have been used to alternately argue for a sister relationship between *P. chiapensis* and *P. strobus*, from eastern North America, or between *P. chiapensis* and *P. monticola*, from western North America [47]. Here, 22 of 24 trees in Figure 7 grouped *P. chiapensis* and *P. strobus* as sister taxa, mostly with reasonably high bootstrap support. When phylogenetic inference strategies were averaged over either gene tree methods (Figure 8) or species tree methods (Figure 9), the {*P. chiapensis*, *P. strobus*} clade was always recovered.

The close phylogenetic affiliation of *P. ayacahuite*, *P. flexilis*, and *P. strobiformis* has long been suspected, as these three species represent similar forms that are continuously distributed from southern British Columbia and Alberta into Honduras [84]. Here, the {*P. ayacahuite*, *P. flexilis*, *P. strobiformis*} clade is well-supported, although relationships among these three species are less clear. Two possibilities, namely ((*P. flexilis*, *P. strobiformis*), *P. ayacahuite*) and ((*P. ayacahuite*, *P. strobiformis*), *P. flexilis*), appear more likely based on our analysis (Figures 7, 8 and 9). Interestingly, Figure 7 finds that the ((*P. ayacahuite*, *P. strobiformis*), *P. flexilis*) clade is well-supported by all strategies that use M.

Beyond these clades, the full phylogeny of this group of pines remains unclear. Considering the trees inferred in Figure 7, relationships among *P. albicaulis*, *P. lambertiana*, *P. monticola*, and the clades {*P. chiapensis*, *P. strobus*} and {*P. ayacahuite*, *P. flexilis*, *P. strobiformis*} are not stable across inference strategies, and bootstrap support is generally low. We might have expected greater resolution in this study, due to the exhaustive sample of the ingroup, extensive intraspecific sampling, large molecular dataset, and ease of species delimitation (the eight ingroup species include well-defined taxa that are morphologically, ecologically, phenologically, and generally geographically distinct).

We can attribute the lack of resolution in the pine phylogeny to several possible sources. First, the loci in the study were chosen because they amplify across a broad range of taxa from subgenus *Strobus* (only eight of whose members are included here), and might therefore be more slowly evolving and less informative for phylogenetic inference than typical loci. Thus, the size of the dataset might not be indicative of its information content for phylogenetic inference. Second, we have focused on strategies that have been implemented for ease of comparison and have not explored the full collection of available methods (e.g., [6,20,85,86]), nor have we considered such techniques as investigation of different subsets of taxa or loci on the basis of the strategies that we have studied (e.g., [16]). A study with a primary goal of resolving the pine phylogeny might achieve greater resolution through analyses that deviate from our standardized procedure. Third, the speciation events of interest might have occurred fast enough that retention of ancestral polymorphisms, as has been observed elsewhere among conifers [47,48,50,87], might inhibit convergence on a stable, well-supported topology. Further work with more loci or faster-evolving loci will be important for distinguishing among these alternatives.

One caveat for interpreting our results is that except in our analyses based on M, we only considered a single lineage sampled within a species. Information on multiple lineages of the same species can have a significant effect on the performance of species tree inference methods, and many methods can use information on coalescences within and between species as part of the inference process (e.g., [19-21,23,25-27,88-90]). Therefore, it is important to keep in mind that we have used one of a number of potential schemes for sampling individuals within our data, as sampling scheme can have an impact on the efficacy of species tree estimators [15,20,36,91,92].

Another caveat is that some of the datasets were obtained from procedures designed to maximize information content at each locus. These optimization procedures yielded datasets with one sequence sampled per species. Because the sequences within these optimized datasets are no longer randomly sampled within each species, a possible concern is that our results are not representative of random samples. This concern might be warranted when considering the inferred relationships of the various pine species in Figures 7, 8 and 9. However, as the strategies applied to each of these optimized datasets retain their general relationships across datasets (e.g., those that are topologically-based and those that are not strictly topologically-based), the conclusions drawn in this article should also hold for large noncoding datasets. Additionally, it is important to mention that an identical dataset is not used for all strategies considered, and notably, strategies that used M relied on different datasets from those that used ML, MP, and NJ. Although this is a limitation, the clustering pattern suggests it is not a major concern. In our principal components (Figure 4), cluster (Figure 5), and correlation (Figure 5) analyses, though the strategies were split by whether they used M or were either topologically-based or not strictly topologically-based, these three categories do not precisely map onto the different datasets. Therefore, though the dataset varies across the 72 strategies, other factors beyond the difference in datasets are contributing substantially to the difference in results.

Finally, a third caveat is that to maintain a uniform procedure across strategies, we did not estimate the nucleotide substitution model before applying maximum likelihood. This choice might have caused some systematic bias in ML gene tree estimates by overparametrizing the substitution model. However, we found that our inference strategies did not cluster by whether maximum likelihood, maximum parsimony, or neighbor-joining was used (see Figures 4B and 5), suggesting that any systematic bias due to using a general time-reversible substitution model did not drive our observed clustering patterns.

## Conclusions

Based on a collection of two-stage strategies that we have investigated, representing a subset of all available methods, our analyses have highlighted several aspects of phylogenetic inference strategies that enable recommendations for inferring rooted phylogenies from large-scale multilocus data. First, it is beneficial to examine multiple strategies [93], considering some methods that use only topological information (e.g., STAR, RTC, and MDC) and others that also incorporate additional information (e.g., Concatenation, SMRT, and STEAC). If species tree topologies returned by these different classes of species tree construction methods agree, then an investigator can be more confident in the inferred tree topology. Second, estimates should not be based solely on species tree construction methods that appear to be biased toward certain types of topologies (e.g., MDC). Instead, it is preferable to utilize these types of methods in conjunction with other approaches. For example, after obtaining an unbalanced inferred tree from an inference method, if MDC also infers the same unbalanced topology, then we can be more confident that the true species topology is actually unbalanced. Finally, it is best to utilize as much information as is available on individuals at every locus. That is, if multiple individuals are sampled within a species at a given locus, then we should use all available sequence data from the species (*i.e.* as many sampled individuals as possible, as in strategies that are based on M). This point is supported by the observation that clades inferred by M tend to “flow” to other strategies (Figure 6). Based on our findings, we recommend the joint consideration of multiple approaches to estimating species trees that originate in different locations in the space of methods and that exhibit diverse properties in their species tree estimates.

## Availability of supporting data

The data used in this study are available in Additional file 3.

## Competing interests

The authors declare that they have no competing interests.

## Authors’ contributions

MD, JS, and NAR designed the project. DBN, AJE, JS, AL, and RC generated and prepared the data. MD and JS performed the data analysis. MD, JS, and NAR wrote the paper, with contributions from all authors. All authors read and approved the final manuscript.

## Supplementary Material

Additional file 1 GenBank accession numbers for sampled sequences. Click here for file

Additional file 2 Supplementary information on phylogenetic inference strategies, sample collection locations, and Procrustes analysis. Click here for file

Additional file 3 **Zip archive containing data files**$D_{p}$**,**$D_{p,0}$**,**$D_{s}$**,**$D_{s,0}$**, and a description of these files.** Click here for file

## References

[B1] Cranston KA Hurwitz B Ware D Stein L Wing RA Species trees from highly incongruent gene trees in rice Syst Biol 2009 58 489 500 10.1093/sysbio/syp054 20525603

[B2] Song S Liu L Edwards SV Wu S Resolving conflict in eutherian mammal phylogeny using phylogenomics and the multispecies coalescent model Proc Natl Acad Sci USA 2012 109 14942 14947 10.1073/pnas.1211733109 22930817 PMC3443116

[B3] McCormack JE Harvey MG Faircloth BC Crawford NG Glenn TC Brumfield RT A phylogeny of birds based on over 1,500 loci collected by target enrichment and high-throughput sequencing PLoS One 2013 8 e54848 10.1371/journal.pone.0054848 23382987 PMC3558522

[B4] Salichos L Rokas A Inferring ancient divergences requires genes with strong phylogenetic signals Nature 2013 497 327 331 10.1038/nature12130 23657258

[B5] Rannala B Yang Z Phylogenetic inference using whole genomes Annu Rev Genomics Hum Genet 2008 9 217 231 10.1146/annurev.genom.9.081307.164407 18767964

[B6] Degnan JH Rosenberg NA Gene tree discordance, phylogenetic inference and the multispecies coalescent Trends Ecol Evol 2009 24 332 340 10.1016/j.tree.2009.01.009 19307040

[B7] Kubatko LS Degnan JH Inconsistency of phylogenetic estimates from concatenated data under coalescence Syst Biol 2007 56 17 24 10.1080/10635150601146041 17366134

[B8] Degnan JH Rosenberg NA Discordance of species trees with their most likely gene trees PLoS Genet 2006 2 e68 10.1371/journal.pgen.0020068 16733550 PMC1464820

[B9] Degnan JH DeGiorgio M Bryant D Rosenberg NA Properties of consensus methods for estimating species trees from gene trees Syst Biol 2009 58 35 54 10.1093/sysbio/syp008 20525567 PMC2909780

[B10] Wang Y Degnan JH The performance of matrix representation with parsimony for inferring species from gene trees Stat Appl Genet Mol Biol 2011 10 21

[B11] Than CV Rosenberg NA Consistency properties of species tree inference by minimizing deep coalescences J Comput Biol 2011 18 1 15 10.1089/cmb.2010.0102 21210728

[B12] Bryant D Janowitz M, Lapointe FJ, McMorris FR, Mirkin B, Roberts FS A classification of consensus methods for phylogenies BioConsensus 2003 Providence, Rhode Island: American Mathematical Society Press 163 183 *DIMACS Series in Discrete Mathematics and Theoretical Computer Science*

[B13] Gadagkar SR Rosenberg MS Kumar S Inferring species phylogenies from multiple genes: Concatenated sequence tree versus consensus gene tree J Exp Zool B Mol Dev Evol 2005 304 64 74 15593277 10.1002/jez.b.21026

[B14] Huang H Knowles LL What is the danger of the anomaly zone for empirical phylogenetics? Syst Biol 2009 58 527 536 10.1093/sysbio/syp047 20525606

[B15] DeGiorgio M Degnan JH Robustness to divergence time underestimation when inferring species trees from estimated gene trees Syst Biol 2014 63 66 82 10.1093/sysbio/syt059 23988674

[B16] Rokas A Williams BL King N Carroll SB Genome-scale approaches to resolving incongruence in molecular phylogenies Nature 2003 425 798 804 10.1038/nature02053 14574403

[B17] Ané C Larget B Baum DA Smith SD Rokas A Bayesian estimation of concordance factors Mol Biol Evol 2007 24 412 426 17095535 10.1093/molbev/msl170

[B18] Liu L Pearl DK Species trees from gene trees: reconstructing Bayesian posterior distributions of a species phylogeny using estimated gene tree distributions Syst Biol 2007 56 504 514 10.1080/10635150701429982 17562474

[B19] Liu L BEST: Bayesian estimation of species trees under the coalescent model Bioinformatics 2008 24 2542 2543 10.1093/bioinformatics/btn484 18799483

[B20] Heled J Drummond AJ Bayesian inference of species trees from multilocus data Mol Biol Evol 2010 27 570 580 10.1093/molbev/msp274 19906793 PMC2822290

[B21] Liu L Yu L Pearl DK Edwards SV Estimating species phylogenies using coalescence times among sequences Syst Biol 2009 58 468 477 10.1093/sysbio/syp031 20525601

[B22] DeGiorgio M Degnan JH Fast and consistent estimation of species trees using supermatrix rooted triples Mol Biol Evol 2010 27 552 569 10.1093/molbev/msp250 19833741 PMC2877557

[B23] Liu L Yu L Pearl DK Maximum tree: a consistent estimator of the species tree J Math Biol 2010 60 95 106 10.1007/s00285-009-0260-0 19283383

[B24] Liu L Yu L Edwards SV A maximum pseudo-likelihood approach for estimating species trees under the coalescent BMC Evo Biol 2010 10 302 10.1186/1471-2148-10-302 PMC2976751 20937096

[B25] Mossel E Roch S Incomplete lineage sorting: consistent phylogeny estimation from multiple loci IEEE/ACM Trans Comput Biol Bioinform 2010 7 166 171 20150678 10.1109/TCBB.2008.66

[B26] Helmkamp LJ Jewett EM Rosenberg NA Improvements to a class of distance matrix methods for inferring species trees from gene trees J Comput Biol 2012 19 632 649 10.1089/cmb.2012.0042 22697239 PMC3400268

[B27] Jewett EM Rosenberg NA iGLASS: an improvement to the GLASS method for estimating species trees from gene trees J Comput Biol 2012 19 293 315 10.1089/cmb.2011.0231 22216756 PMC3298679

[B28] Huang H He Q Kubatko LS Knowles LL Sources of error inherent in species-tree estimation: impact of mutational and coalescent effects on accuracy and implications for choosing among different methods Syst Biol 2010 59 573 583 10.1093/sysbio/syq047 20833951

[B29] Chung Y Ané C Comparing two Bayesian methods for gene tree/species tree reconstruction: simulations with incomplete lineage sorting and horizontal gene transfer Syst Biol 2011 60 261 275 10.1093/sysbio/syr003 21368324

[B30] Leaché AD Rannala B The accuracy of species tree estimation under simulation: a comparison of methods Syst Biol 2011 60 126 137 10.1093/sysbio/syq073 21088009

[B31] Swenson MS Suri R Linder CR Warnow T An experimental study of Quartets MaxCut and other supertree methods Algorithms Mol Biol 2011 6 7 10.1186/1748-7188-6-7 21504600 PMC3101644

[B32] Yang J Warnow T Fast and accurate methods for phylogenomic analyses BMC Bioinformatics 2011 12 Suppl9 S4 22152123 10.1186/1471-2105-12-S9-S4 PMC3283310

[B33] Jennings WB Edwards SV Speciational history of Australian grass finches (*Poephila*) inferred from thirty gene trees Evolution 2005 59 2033 2047 16261740

[B34] Brumfield RT Liu L Lum DE Edwards SV Comparison of species tree methods for reconstructing the phylogeny of bearded manakins (Aves: Pipridae, *Manacus*) from multilocus sequence data Syst Biol 2008 57 719 731 10.1080/10635150802422290 18853359

[B35] Carling MD Brumfield RT Integrating phylogenetic and population genetic analyses of multiple loci to test species divergence hypotheses in *Passerina* buntings Genetics 2008 178 363 377 10.1534/genetics.107.076422 18202379 PMC2206085

[B36] Liu L Pearl DK Brumfield RT Edwards SV Estimating species trees using multiple-allele DNA sequence data Evolution 2008 62 2080 2091 10.1111/j.1558-5646.2008.00414.x 18462214

[B37] Carstens BC Knowles LL Estimating species phylogeny from gene-tree probabilities despite incomplete lineage sorting: an example from *Melanoplus* grasshoppers Syst Biol 2007 56 400 411 10.1080/10635150701405560 17520504

[B38] Linnen CR Farrell BD Comparison of methods for species-tree inference in the sawfly genus *Neodiprion* (Hymenoptera: Diprionidae) Syst Biol 2008 57 876 890 10.1080/10635150802580949 19085330

[B39] Espregueira Themudo G Wielstra B Arntzen JW Multiple nuclear and mitochondrial genes resolve the branching order of a rapid radiation of crested newts (Triturus, Salamandridae) Mol Phylogenet Evol 2009 52 321 328 10.1016/j.ympev.2009.03.024 19348957

[B40] Buerki S Forest F Salamin N Alvarez N Comparative performance of supertree algorithms in large data sets using the soapberry family (Sapindaceae) as a case study Syst Biol 2011 60 32 44 10.1093/sysbio/syq057 21068445

[B41] Takezaki N Nei M Empirical tests of the reliability of phylogenetic trees constructed with microsatellite DNA Genetics 2008 178 385 392 10.1534/genetics.107.081505 18202381 PMC2206087

[B42] Hird S Kubatko L Carstens B Rapid and accurate species tree estimation for phylogenetic investigations using replicated subsampling Mol Phylogenet Evol 2010 57 888 898 10.1016/j.ympev.2010.08.006 20727977

[B43] Belfiore NM Liu L Moritz C Multilocus phylogenetics of a rapid radiation in the genus *Thomomys* (Rodentia: Geomyidae) Syst Biol 2008 57 294 310 10.1080/10635150802044011 18432550

[B44] Kubatko LS Gibbs HL Bloomquist EW Inferring species-level phylogenies and taxonomic distinctiveness using multilocus data in *Sistrus* rattlesnakes Syst Biol 2011 60 393 409 10.1093/sysbio/syr011 21389297

[B45] Gatesy J Baker RH Hidden likelihood support in genomic data: can forty-five wrongs make a right? Syst Biol 2005 54 483 492 10.1080/10635150590945368 16012113

[B46] Edwards SV Liu L Pearl DK High-resolution species trees without concatenation Proc Natl Acad Sci USA 2007 104 5936 5941 10.1073/pnas.0607004104 17392434 PMC1851595

[B47] Syring J Farrell K Businský R Cronn R Liston A Widespread genealogical nonmonophyly in species of *Pinus* subgenus *Strobus* Syst Biol 2007 56 163 181 10.1080/10635150701258787 17454973

[B48] Bouillé M Bousquet J Trans-species shared polymorphisms at orthologous nuclear gene loci among distant species in the conifer *Picea* (Pinaceae): implications for the long-term maintenance of genetic diversity in trees Am J Bot 2005 92 63 73 10.3732/ajb.92.1.63 21652385

[B49] Ma XF Szmidt AE Wang XR Genetic structure and evolutionary history of a diploid hybrid pine *Pinus densata* inferred from the nucleotide variation at seven gene loci Mol Biol Evol 2006 23 807 816 10.1093/molbev/msj100 16446291

[B50] Willyard A Cronn R Liston A Reticulate evolution and incomplete lineage sorting among the ponderosa pines Mol Phylogenet Evol 2009 52 498 511 10.1016/j.ympev.2009.02.011 19249377

[B51] Savolainen O Pyhäjärvi T Genomic diversity in forest trees Curr Opin Plant Biol 2007 10 162 167 10.1016/j.pbi.2007.01.011 17292660

[B52] Kral R Flora of North America editorial committee F, Flora of North America editorial committee F *Pinus* Flora of North America (North of Mexico) 1993 New York: Oxford University Press 373 398

[B53] Perry JP The Pines of Mexico and Central America 1991 Portland: Timber Press

[B54] Syring J Willyard A Cronn R Liston A Evolutionary relationships among *Pinus* (Pinaceae) subsections inferred from multiple low-copy nuclear loci Am J Bot 2005 92 2086 2100 10.3732/ajb.92.12.2086 21646125

[B55] Eckert AJ van Heerwaarden J Wegrzyn JL Nelson CD Ross-Ibarra J González-Martínez SC Neale DB Patterns of population structure and environmental associations to aridity across the range of Loblolly pine (*Pinus taeda* L., Pinaceae) Genetics 2010 185 969 982 10.1534/genetics.110.115543 20439779 PMC2907212

[B56] Wegrzyn JL Lee JM Liechty J Neale DB PineSAP—sequence alignment and SNP identification pipeline Bioinformatics 2009 25 2609 2610 10.1093/bioinformatics/btp477 19667082 PMC2752621

[B57] Ewing B Hillier L Wendl MC Green P Base-calling of automated sequencer traces using phred. I. Accuracy assessment Genome Res 1998 8 175 185 10.1101/gr.8.3.175 9521921

[B58] Lee WH Vega VB Heterogeneity detector: finding heterogeneous positions in Phred/Phrap assemblies Bioinformatics 2004 20 2863 2864 10.1093/bioinformatics/bth301 15130927

[B59] Edgar RC MUSCLE: a multiple sequence alignment method with reduced time and space complexity BMC Bioinformatics 2004 5 113 10.1186/1471-2105-5-113 15318951 PMC517706

[B60] Edgar RC MUSCLE: multiple sequence alignment with high accuracy and high throughput Nucleic Acids Res 2004 32 1792 1797 10.1093/nar/gkh340 15034147 PMC390337

[B61] Ewing B Green P Base-calling of automated sequencer traces using phred. II. Error probabilities Genome Res 1998 8 186 194 9521922

[B62] Parks M Cronn R Liston A Increasing phylogenetic resolution at low taxonomic levels using massively parallel sequencing of chloroplast genomes BMC Biol 2009 7 84 10.1186/1741-7007-7-84 19954512 PMC2793254

[B63] Felsenstein J Inferring Phylogenies 2004 Sunderland, MA: Sinauer Associates

[B64] Felsenstein J Confidence limits on phylogenies: an approach using the bootstrap Evolution 1985 39 783 791 10.2307/2408678 28561359

[B65] Efron B Tibshirani RJ An Introduction to the Bootstrap 1993 New York: Chapman and Hall

[B66] Swofford DL PAUP*. Phylogenetic analysis Using Parsimony (* and Other Methods). Version 4 2003 Sunderland, MA: Sinauer Associates

[B67] de Queiroz A Gatesy J The supermatrix approach to systematics Trends Ecol Evol 2007 22 34 41 10.1016/j.tree.2006.10.002 17046100

[B68] Ewing GB Ebersberger I Schmidt HA von Haeseler A Rooted triple consensus and anomalous gene trees BMC Evol Biol 2008 8 118 18439266 10.1186/1471-2148-8-118 PMC2409437

[B69] Maddison WP Gene trees in species trees Syst Biol 1997 46 523 536 10.1093/sysbio/46.3.523

[B70] Than C Nakhleh L Species tree inference by minimizing deep coalescences PLoS Comput Biol 2009 5 e1000501 10.1371/journal.pcbi.1000501 19749978 PMC2729383

[B71] Page RDM Guigó R, Gusfield D Modified mincut supertrees Algorithms in Bioinformatics, Second International Workshop, WABI, 2002, Rome, Italy, September 17–21, 2002, Proceedings (Lecture Notes in Computer Science Vol. 2452) 2002 Berlin: Springer 537 552

[B72] Strimmer K von Haeseler A Quartet puzzling: a quartet maximum-likelihood method for reconstructing tree topologies Mol Biol Evol 1996 13 964 969 10.1093/oxfordjournals.molbev.a025664

[B73] Robinson DR Foulds LR Comparison of phylogenetic trees Math Biosci 1981 53 131 147 10.1016/0025-5564(81)90043-2

[B74] Dryden IL Mardia KV Statistical Shape Analysis 1998 Chichester: Wiley

[B75] Cox TF Cox MAA Multidimensional Scaling, 2nd edition 2001 Boca Raton: Chapman and Hall

[B76] Gower JC Dijksterhuis GB Procrustes Problems 2004 New York: Oxford University Press

[B77] Ward JH Hierarchical grouping to optimize an objective function J Am Stat Assoc 1963 58 236 244 10.1080/01621459.1963.10500845

[B78] Sackin MJ “Good” and “bad” phenograms Syst Zool 1972 21 225 226 10.2307/2412292

[B79] Colless DH Phylogenetics, the theory and practice of phylogenetic systematics (book review) Syst Zool 1982 31 100 104 10.2307/2413420

[B80] Shao KT Sokal RR Tree balance Syst Zool 1990 39 266 276 10.2307/2992186

[B81] Kirkpatrick M Slatkin M Searching for evolutionary patterns in the shape of a phylogenetic tree Evolution 1993 47 1171 1181 10.2307/2409983 28564277

[B82] Conrad DF Jakobsson M Coop G Wen X Wall JD Rosenberg NA Pritchard JK A worldwide survey of haplotype variation and linkage disequilibrium in the human genome Nat Genet 2006 38 1251 1260 10.1038/ng1911 17057719

[B83] Than CV Rosenberg NA Mathematical properties of the deep coalescence cost IEEE/ACM Trans Comput Biol Biosci 2013 10 61 72 10.1109/TCBB.2012.133 23702544

[B84] Critchfield WB Little EL Jr Geographic Distribution of the Pines of the World 1966 Washington, DC: USDA Forest Service Miscellaneous Publication 991

[B85] Liu L Yu L Kubatko L Pearl DK Edwards SV Coalescent methods for estimating phylogenetic trees Mol Phylogenet Evol 2009 53 320 328 10.1016/j.ympev.2009.05.033 19501178

[B86] Knowles LL Kubatko LS Estimating Species Trees: Practical and Theoretical Aspects 2010 Hoboken: Wiley-Blackwell

[B87] Syring J del Castillo RF Cronn R Liston A Multiple nuclear loci reveal the distinctiveness of the threatened neotropical pine *Pinus chiapensis* Syst Bot 2007 32 703 717 10.1600/036364407783390836

[B88] Kubatko LS Carstens BC Knowles LL STEM: species tree estimation using maximum likelihood for gene trees under coalescence Bioinformatics 2009 25 971 973 10.1093/bioinformatics/btp079 19211573

[B89] Liu L Yu L Estimating species trees from unrooted gene trees Syst Biol 2012 60 661 667 21447481 10.1093/sysbio/syr027

[B90] Wu Y Coalescent-based species tree inference from gene tree topologies under incomplete lineage sorting by maximum likelihood Evolution 2012 66 763 775 10.1111/j.1558-5646.2011.01476.x 22380439

[B91] McCormack JE Huang H Knowles LL Maximum likelihood estimates of species trees: how accuracy of phylogenetic inference depends upon the divergence history and sampling design Syst Biol 2009 58 501 508 10.1093/sysbio/syp045 20525604

[B92] Camargo A Avila LJ Morando M Sites JW Jr Accuracy and precision of species trees: effects of locus, individual, and base pair sampling on inference of species trees in lizards of the *Liolaemus darwinii* group (Squamata, Liolaemidae) Syst Biol 2012 61 272 288 10.1093/sysbio/syr105 22076301

[B93] Anisimova M Liberles DA Philippe H Provan J Pupko T von Haeseler A State-of the art methodologies dictate new standards for phylogenetic analysis BMC Evol Biol 2013 13 161 23914788 10.1186/1471-2148-13-161 PMC3751533

