# Supplementary material for: An empirical evaluation of two-stage species tree inference strategies using a multilocus dataset from North American pines
Source: BMC Evol Biol. 2014 Mar 29;14:67. doi: 10.1186/1471-2148-14-67 (PMC4021425; doi:10.1186/1471-2148-14-67)
Supplement: Additional file 2 — Supplementary information on phylogenetic inference strategies, sample collection locations, and Procrustes analysis. [file 1471-2148-14-67-S2.pdf]

Supplementary material: an empirical evaluation of two-stage species tree inference strategies using a multilocus dataset from North American pines

Michael DeGiorgio[1], John Syring[3], Andrew J. Eckert[4], Aaron I. Liston[5], Richard Cronn[6], David B. Neale[7], and Noah A. Rosenberg[8]

November 25, 2013

Table S1: Phylogenetic inference strategies

Index	Species tree inference method	Gene tree inference method	Outgroup species	Concatenation or consensus	Strictly topology-based
1	Concatenation	ML	*P. gerardiana*	Concatenation	No
2	Concatenation	ML	*P. bungeana*	Concatenation	No
3	Concatenation	ML	*P. squamata*	Concatenation	No
4	Concatenation	MP	*P. gerardiana*	Concatenation	No
5	Concatenation	MP	*P. bungeana*	Concatenation	No
6	Concatenation	MP	*P. squamata*	Concatenation	No
7	Concatenation	NJ	*P. gerardiana*	Concatenation	No
8	Concatenation	NJ	*P. bungeana*	Concatenation	No
9	Concatenation	NJ	*P. squamata*	Concatenation	No
10	Concatenation	M	*P. gerardiana*	Concatenation	No
11	Concatenation	M	*P. bungeana*	Concatenation	No
12	Concatenation	M	*P. squamata*	Concatenation	No
13	SMRT	ML	*P. gerardiana*	Concatenation	No
14	SMRT	ML	*P. bungeana*	Concatenation	No
15	SMRT	ML	*P. squamata*	Concatenation	No
16	SMRT	MP	*P. gerardiana*	Concatenation	No
17	SMRT	MP	*P. bungeana*	Concatenation	No
18	SMRT	MP	*P. squamata*	Concatenation	No
19	SMRT	NJ	*P. gerardiana*	Concatenation	No
20	SMRT	NJ	*P. bungeana*	Concatenation	No
21	SMRT	NJ	*P. squamata*	Concatenation	No
22	SMRT	M	*P. gerardiana*	Concatenation	No
23	SMRT	M	*P. bungeana*	Concatenation	No
24	SMRT	M	*P. squamata*	Concatenation	No
25	STEAC	ML	*P. gerardiana*	Consensus	No
26	STEAC	ML	*P. bungeana*	Consensus	No
27	STEAC	ML	*P. squamata*	Consensus	No
28	STEAC	MP	*P. gerardiana*	Consensus	No
29	STEAC	MP	*P. bungeana*	Consensus	No
30	STEAC	MP	*P. squamata*	Consensus	No
31	STEAC	NJ	*P. gerardiana*	Consensus	No
32	STEAC	NJ	*P. bungeana*	Consensus	No
33	STEAC	NJ	*P. squamata*	Consensus	No
34	STEAC	M	*P. gerardiana*	Consensus	No
35	STEAC	M	*P. bungeana*	Consensus	No
36	STEAC	M	*P. squamata*	Consensus	No
37	STAR	ML	*P. gerardiana*	Consensus	Yes
38	STAR	ML	*P. bungeana*	Consensus	Yes
39	STAR	ML	*P. squamata*	Consensus	Yes
40	STAR	MP	*P. gerardiana*	Consensus	Yes
41	STAR	MP	*P. bungeana*	Consensus	Yes
42	STAR	MP	*P. squamata*	Consensus	Yes
43	STAR	NJ	*P. gerardiana*	Consensus	Yes
44	STAR	NJ	*P. bungeana*	Consensus	Yes
45	STAR	NJ	*P. squamata*	Consensus	Yes
46	STAR	M	*P. gerardiana*	Consensus	Yes
47	STAR	M	*P. bungeana*	Consensus	Yes
48	STAR	M	*P. squamata*	Consensus	Yes
49	RTC	ML	*P. gerardiana*	Consensus	Yes
50	RTC	ML	*P. bungeana*	Consensus	Yes
51	RTC	ML	*P. squamata*	Consensus	Yes
52	RTC	MP	*P. gerardiana*	Consensus	Yes
53	RTC	MP	*P. bungeana*	Consensus	Yes
54	RTC	MP	*P. squamata*	Consensus	Yes
55	RTC	NJ	*P. gerardiana*	Consensus	Yes
56	RTC	NJ	*P. bungeana*	Consensus	Yes
57	RTC	NJ	*P. squamata*	Consensus	Yes
58	RTC	M	*P. gerardiana*	Consensus	Yes
59	RTC	M	*P. bungeana*	Consensus	Yes
60	RTC	M	*P. squamata*	Consensus	Yes
61	MDC	ML	*P. gerardiana*	Consensus	Yes
62	MDC	ML	*P. bungeana*	Consensus	Yes
63	MDC	ML	*P. squamata*	Consensus	Yes
64	MDC	MP	*P. gerardiana*	Consensus	Yes
65	MDC	MP	*P. bungeana*	Consensus	Yes
66	MDC	MP	*P. squamata*	Consensus	Yes
67	MDC	NJ	*P. gerardiana*	Consensus	Yes
68	MDC	NJ	*P. bungeana*	Consensus	Yes
69	MDC	NJ	*P. squamata*	Consensus	Yes
70	MDC	M	*P. gerardiana*	Consensus	Yes
71	MDC	M	*P. bungeana*	Consensus	Yes
72	MDC	M	*P. squamata*	Consensus	Yes

TABLE S2. Sampled *Pinus* subsection *Strobus* (ingroup) and *Gerardianae* (outgroup) representatives. Dashes indicate missing data.

Sample id	Species	Subsection	State[a]	Country	Latitude	Longitude
Pial.ao	*P. albicaulis*	*Strobus*	---	---	---	---
Pial-2	*P. albicaulis*	*Strobus*	British Columbia	Canada	54.880	-125.374
Pial-3	*P. albicaulis*	*Strobus*	Alberta	Canada	53.707	-119.607
Pial-4	*P. albicaulis*	*Strobus*	British Columbia	Canada	50.095	-122.899
Pial-5	*P. albicaulis*	*Strobus*	British Columbia	Canada	49.370	-119.921
Pial-6	*P. albicaulis*	*Strobus*	Alberta	Canada	49.366	-114.252
Pial-7	*P. albicaulis*	*Strobus*	Washington	USA	48.707	-118.472
Pial-8	*P. albicaulis*	*Strobus*	Washington	USA	48.311	-120.565
Pial-9	*P. albicaulis*	*Strobus*	Washington	USA	47.821	-123.134
Pial-10	*P. albicaulis*	*Strobus*	Washington	USA	46.244	-121.474
Pial-12	*P. albicaulis*	*Strobus*	Montana	USA	45.042	-109.451
Pial-13	*P. albicaulis*	*Strobus*	Oregon	USA	44.723	-118.575
Pial-15	*P. albicaulis*	*Strobus*	Montana	USA	44.543	-111.409
Pial-16	*P. albicaulis*	*Strobus*	California	USA	41.798	-122.158
Pial-17	*P. albicaulis*	*Strobus*	Oregon	USA	43.380	-121.570
Piay-1	*P. ayacahuite*[b]	*Strobus*	La Paz	Honduras	14.117	-87.817
Piay-2	*P. ayacahuite*[b]	*Strobus*	Ocotepeque	Honduras	14.417	-89.133
Piay-3	*P. ayacahuite*[b]	*Strobus*	Totonicapan	Guatemala	14.933	-91.350
Piay-4	*P. ayacahuite*[b]	*Strobus*	Chiapas	Mexico	16.617	-92.367
Piay-5	*P. ayacahuite*[b]	*Strobus*	Oaxaca	Mexico	17.150	-97.633
Piay-6	*P. ayacahuite*[c]	*Strobus*	Puebla	Mexico	19.167	-98.583
Piay-7	*P. ayacahuite*[d]	*Strobus*	Estado de Mexico	Mexico	19.167	-98.800
Piay-8	*P. ayacahuite*[c]	*Strobus*	Michoacan	Mexico	19.600	-102.330
Piay-9	*P. ayacahuite*[c]	*Strobus*	Jalisco	Mexico	20.383	-105.067
Piay-10	*P. ayacahuite*[c]	*Strobus*	Aguascalientes	Mexico	19.600	-99.767
Piay-13	*P. ayacahuite*[d]	*Strobus*	Coahuila	Mexico	25.233	-100.433
Piay-14	*P. ayacahuite*[b]	*Strobus*	Chiapas	Mexico	15.417	-92.333
Piay-15	*P. ayacahuite*[b]	*Strobus*	Oaxaca	Mexico	17.400	-96.483
Piay-16	*P. ayacahuite*[b]	*Strobus*	Chiapas	Mexico	16.300	-93.283
Piay-17	*P. ayacahuite*[b]	*Strobus*	Huehuetenango	Guatemala	15.733	-91.458
Piay-18	*P. ayacahuite*[d]	*Strobus*	Nuevo Leon	Mexico	25.233	-100.433
Piay-19	*P. ayacahuite*[d]	*Strobus*	Nuevo Leon	Mexico	25.883	-100.217
Pist-16	*P. chiapensis*	*Strobus*	---	Mexico	---	---
Pist-17	*P. chiapensis*	*Strobus*	Huehuetenango	Guatemala	15.800	-91.300
Pist-18	*P. chiapensis*	*Strobus*	Oaxaca	Mexico	17.217	-97.983
Pifl-1	*P. flexilis*	*Strobus*	British Columbia	Canada	50.000	-115.500
Pifl-2	*P. flexilis*	*Strobus*	Alberta	Canada	52.254	-116.397
Pifl-3	*P. flexilis*	*Strobus*	Alberta	Canada	49.083	-114.230
Pifl-4	*P. flexilis*	*Strobus*	Utah	USA	37.658	-112.708
Pifl-5	*P. flexilis*	*Strobus*	Utah	USA	37.017	-110.867
Pifl-8	*P. flexilis*	*Strobus*	Nevada	USA	36.333	-115.683
Pifl-9	*P. flexilis*	*Strobus*	New Mexico	USA	36.333	-105.667
Pifl-10	*P. flexilis*	*Strobus*	New Mexico	USA	35.216	-106.450
Pifl-11	*P. flexilis*	*Strobus*	Colorado	USA	38.733	-104.950
Pifl-12	*P. flexilis*	*Strobus*	Colorado	USA	37.650	-106.650
Pifl-13	*P. flexilis*	*Strobus*	Colorado	USA	37.367	-108.067
Pifl-14	*P. flexilis*	*Strobus*	Colorado	USA	36.490	-106.367
Pifl-15	*P. flexilis*	*Strobus*	California	USA	36.383	-118.183
Pifl-16	*P. flexilis*	*Strobus*	Colorado	USA	---	---
Pifl-17	*P. flexilis*	*Strobus*	Colorado	USA	---	---
Pila-17	*P. lambertiana*	*Strobus*	California	USA	41.687	-123.831
Pila-18	*P. lambertiana*	*Strobus*	California	USA	39.494	-122.640
Pila-19	*P. lambertiana*	*Strobus*	California	USA	35.468	-115.692
Pila-20	*P. lambertiana*	*Strobus*	California	USA	34.429	-115.193
Pila-21	*P. lambertiana*	*Strobus*	California	USA	36.790	-118.929
Pila-22	*P. lambertiana*	*Strobus*	California	USA	35.478	-118.599
Pila-23	*P. lambertiana*	*Strobus*	Oregon	USA	42.450	-120.680

Pila-24	*P. lambertiana*	*Strobus*	California	USA	34.606	-119.309
Pila-26	*P. lambertiana*	*Strobus*	Baja California	Mexico	31.000	-115.500
Pila-27	*P. lambertiana*	*Strobus*	California	USA	41.473	-121.159
Pila-28	*P. lambertiana*	*Strobus*	Oregon	USA	44.980	-121.850
Pila-29	*P. lambertiana*	*Strobus*	California	USA	---	---
Pimn-1	*P. monticola*	*Strobus*	California	USA	41.565	-121.517
Pimn-2	*P. monticola*	*Strobus*	California	USA	---	---
Pimn-3	*P. monticola*	*Strobus*	California	USA	38.975	-120.284
Pimn-4	*P. monticola*	*Strobus*	California	USA	36.046	-118.349
Pimn-5	*P. monticola*	*Strobus*	California	USA	---	---
Pimn-6	*P. monticola*	*Strobus*	California	USA	---	---
Pimn-7	*P. monticola*	*Strobus*	California	USA	---	---
Pimn-8	*P. monticola*	*Strobus*	California	USA	39.266	-120.242
Pimn-9	*P. monticola*	*Strobus*	Oregon	USA	42.033	-122.942
Pimn-10	*P. monticola*	*Strobus*	California	USA	---	---
Pimn-11	*P. monticola*	*Strobus*	Washington	USA	48.736	-117.220
Pimn-12	*P. monticola*	*Strobus*	Oregon	USA	42.372	-124.290
Pimn-13	*P. monticola*	*Strobus*	Oregon	USA	45.131	-111.683
Pimn-14	*P. monticola*	*Strobus*	Washington	USA	46.150	-122.000
Pimn-15	*P. monticola*	*Strobus*	Washington	USA	47.400	-123.000
Pimn-16	*P. monticola*	*Strobus*	Oregon	USA	44.260	-122.000
Pimn-17	*P. monticola*	*Strobus*	Idaho	USA	47.250	-116.000
Pimn-18	*P. monticola*	*Strobus*	Montana	USA	48.367	-113.933
Pisb-1	*P. strobiformis*	*Strobus*	Colorado	USA	37.417	-107.000
Pisb-2	*P. strobiformis*	*Strobus*	Arizona	USA	37.282	-110.821
Pisb-3	*P. strobiformis*	*Strobus*	New Mexico	USA	35.917	-108.667
Pisb-4	*P. strobiformis*	*Strobus*	Arizona	USA	35.317	-111.700
Pisb-5	*P. strobiformis*	*Strobus*	New Mexico	USA	35.142	-108.145
Pisb-6	*P. strobiformis*	*Strobus*	Arizona	USA	34.330	-111.417
Pisb-7	*P. strobiformis*	*Strobus*	Arizona	USA	34.108	-109.567
Pisb-8	*P. strobiformis*	*Strobus*	New Mexico	USA	33.833	-108.833
Pisb-9	*P. strobiformis*	*Strobus*	New Mexico	USA	33.167	-107.467
Pisb-10	*P. strobiformis*	*Strobus*	New Mexico	USA	32.917	-105.750
Pisb-11	*P. strobiformis*	*Strobus*	New Mexico	USA	32.750	-107.583
Pisb-12	*P. strobiformis*	*Strobus*	Arizona	USA	32.667	-109.867
Pisb-13	*P. strobiformis*	*Strobus*	Arizona	USA	32.443	-110.788
Pisb-14	*P. strobiformis*[e]	*Strobus*	Coahuila	Mexico	25.233	-100.433
Pisb-15	*P. strobiformis*[e]	*Strobus*	Nuevo Leon	Mexico	24.883	-100.217
Pisb-16	*P. strobiformis*	*Strobus*	New Mexico	USA	32.800	-105.800
Pisb-17	*P. strobiformis*	*Strobus*	Arizona	USA	34.447	-111.358
Pisb-18	*P. strobiformis*	*Strobus*	Nuevo Leon	Mexico	24.883	-100.217
Pist-1	*P. strobus*	*Strobus*	Newfoundland and Labrador	Canada	48.520	-54.200
Pist-2	*P. strobus*	*Strobus*	Ontario	Canada	47.530	-83.100
Pist-3	*P. strobus*	*Strobus*	Minnesota	USA	47.402	-94.438
Pist-4	*P. strobus*	*Strobus*	Quebec	Canada	47.160	-72.580
Pist-5	*P. strobus*	*Strobus*	New Brunswick	Canada	46.480	-66.070
Pist-6	*P. strobus*	*Strobus*	Michigan	USA	46.377	-84.657
Pist-7	*P. strobus*	*Strobus*	Wisconsin	USA	46.108	-90.944
Pist-8	*P. strobus*	*Strobus*	Ontario	Canada	45.590	-77.230
Pist-9	*P. strobus*	*Strobus*	Maine	USA	44.580	-67.050
Pist-10	*P. strobus*	*Strobus*	Nova Scotia	Canada	44.410	-65.220
Pist-11	*P. strobus*	*Strobus*	Vermont	USA	44.290	-73.060
Pist-12	*P. strobus*	*Strobus*	Wisconsin	USA	42.988	-90.668
Pist-13	*P. strobus*	*Strobus*	New York	USA	42.150	-76.450
Pist-14	*P. strobus*	*Strobus*	Pennsylvania	USA	41.100	-82.150
Pist-15	*P. strobus*	*Strobus*	North Carolina	USA	35.800	-82.700
Pist-19	*P. strobus*	*Strobus*	Pennsylvania	USA	---	---
Pibu-1	*P. bungeana*	*Gerardianae*	---	---	---	---
Pige-1	*P. gerardiana*	*Gerardianae*	---	---	---	---
Pige-2	*P. gerardiana*	*Gerardianae*	Gilgit	Pakistan	35.386	74.838
Pisq-1	*P. squamata*	*Gerardianae*	NE Yunnan	China	26.869	103.013

Pisq-2	*P. squamata*	*Gerardianae*	NE Yunnan	China	26.87	103.014

[a]State, province or department depending on the country of origin.
[b]*P. ayacahuite* var. *ayacahuite*
[c]*P. ayacahuite* var. *veitchii*
[d]*P. ayacahuite* var. *brachyptera*
[e]Putatively *P. ayacahuite* var. *brachyptera*

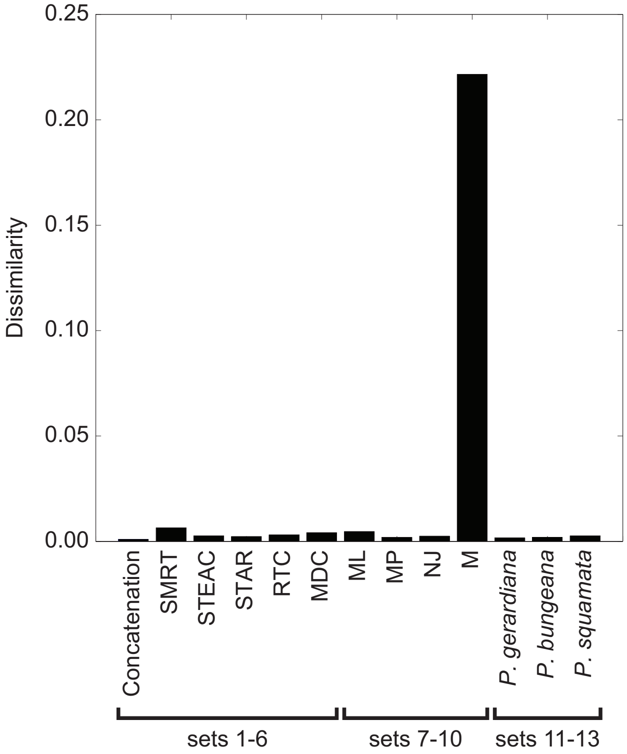

Figure S1: Procrustes analysis of the influence of specific features of phylogenetic strategies on principal component plots. Principal components analysis of phylogenetic inference strategies was performed as in Figure 4 with the exception that it was applied to each of 13 subsets $\mathcal{F}_1, \mathcal{F}_2, \ldots, \mathcal{F}_{13}$ of phylogenetic inference strategies for which one feature of a strategy was removed. $\mathcal{F}_1, \mathcal{F}_2, \ldots, \mathcal{F}_6$ are subsets containing 60 strategies, in which one of the six species tree construction methods is not included, $\mathcal{F}_7, \mathcal{F}_8, \ldots, \mathcal{F}_{10}$ are subsets containing 54 strategies, in which one of the four gene tree inference methods is not included, and $\mathcal{F}_{11}, \mathcal{F}_{12}, \mathcal{F}_{13}$ are subsets containing 48 strategies, in which one of the three outgroup species is not included. In other words, we performed principal components analysis on 13 different datasets: six in which a species tree construction method was removed (Concatenation, SMRT, STEAC, STAR, RTC, and MDC), four in which a gene tree inference method was removed (ML, MP, NJ, and M), and three in which an outgroup was removed (*P. gerardiana*, *P. bungeana*, and *P. squamata*). The results of each principal components analysis were projected into the two-dimensional plane spanned by their first and second principal components. The points in each two-dimensional plane were compared to the points in Figure 4*A* through Procrustes analysis. Each comparison gives a dissimilarity score computed using eq. 3.
